# Gravity‐Tolerant In‐Flight 3D Bioprinting Enabled by Stereolithography for Space Tissue Engineering

**DOI:** 10.1002/advs.202520715

**Published:** 2026-01-21

**Authors:** Bianca Lemke, Matthias R. Kollert, Tobias Lam, Tobias Thiele, Nicolas Göbel, Lisa R. Köhn, Gabriela Korus, Lutz Kloke, Georg N. Duda

**Affiliations:** ^1^ Julius Wolff Institut Berlin Institute of Health at Charité ‐ Universitätsmedizin Berlin Berlin Germany; ^2^ BIH Center for Regenerative Therapies Berlin Institute of Health at Charité ‐ Universitätsmedizin Berlin Berlin Germany; ^3^ Berlin School for Regenerative Therapies Charité ‐ Universitätsmedizin Berlin Berlin Germany; ^4^ Cellbricks GmbH Berlin Germany

**Keywords:** biofabrication, in‐space manufacturing, microgravity, parabolic flight, tissue engineering

## Abstract

Growing efforts toward long‐duration human space missions demand novel strategies for the treatment of acute injuries in extreme environments. Technologies for space applications must perform reliably even under environmental stressors rarely encountered on Earth, such as gravitational fluctuations. However, space‐compatible personalized therapy approaches for treatment of high‐risk injuries remain scarce. 3D bioprinting represents an advanced technology with promising potential to address this unmet medical need by enabling the on‐demand fabrication of patient‐specific tissue constructs for regenerative wound care. Here, the robust 3D printing of both acellular and cell‐laden hydrogel constructs is demonstrated using photo‐ and bioinks under diverse environmental conditions, including microgravity and hypergravity phases encountered during parabolic flight (0–1.8 g). Despite dynamic accelerative conditions, the developed flight‐compliant, closed stereolithographic (SLA) bioprinting system successfully printed 3D structures with maintained dimensional fidelity. Irrespective of gravitational forces, high cell viability was preserved in both fibroblast‐ and keratinocyte‐laden constructs. High‐resolution features are achieved with precision comparable to normal‐gravity controls. Complex architectures, including gyroids, can be fabricated with smooth, continuous surfaces. These findings establish SLA bioprinting as a robust and gravity‐tolerant platform for fabricating viable, cell‐laden constructs—offering a promising pathway for advancing tissue engineering in space and in extreme conditions on Earth.

## Graphical abstract

A flight‐compliant, closed stereolithographic 3D bioprinting system enables robust fabrication of acellular and cell‐laden hydrogel constructs under varying gravitational conditions, including microgravity and hypergravity phases. Printed structures retain dimensional fidelity and high‐resolution features, while cell viability remains high, establishing a gravity‐tolerant platform for tissue engineering in space and other extreme environments.

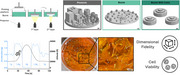

## 1 Introduction

In space exploration, medical capabilities are extremely limited [1]. Similar challenges exist in remote and underserved regions, the lack of advanced medical technologies contributes to higher mortality and disability rates [2, 3]. As humanity advances toward long‐term, deep‐space missions and extraterrestrial colonization [1], the basic medical technologies currently available onboard may be insufficient to provide effective treatment for potentially life‐threatening injuries in space [4, 5]. Bioprinting represents an advanced medical technology that promises the fabrication of living tissue analogues which can replace injured tissue or promote functional regeneration, harnessing decades of tissue engineering research to enable clinically relevant solutions [6, 7]. Recent advancements indicate that bioprinting could also offer a feasible pathway toward autonomous fabrication of living tissue analogues in the context of space exploration, as it is a compact and resource‐efficient technology with the potential for further automation [8, 9, 10]. Bioprinting techniques are broadly categorized into extrusion‐based and vat photopolymerization methods, the latter comprising stereolithography (SLA), including its subtype digital light processing (DLP), and volumetric additive manufacturing (e.g. xolography) [11, 12, 13]. Common to all techniques is the use of hydrogel‐based bioinks – polymer solutions that support maintenance and processing of living cells – to precisely assemble complex 3D structures [11].

In hydrogel‐based bioprinting, cell sedimentation is a known challenge [7, 14], which could be mitigated for example by active ink mixing or by tailoring ink properties like viscosity [15, 16].

The ability to spatially control microenvironment organization and cell placement makes bioprinting a key technology for generating in vitro models (stem cell maintenance, tumor environments [17]) across multiple tissue types and biologically viable constructs suitable for tissue regeneration (organ replacement for transplantation, local drug delivery) [11, 18]. Combining versatility and precision, bioprinting promises biomaterial‐based solutions tailored to patient‐specific medical needs, both in terms of spatial arrangement and architecture as well as biological composition and function.

**FIGURE 1:**
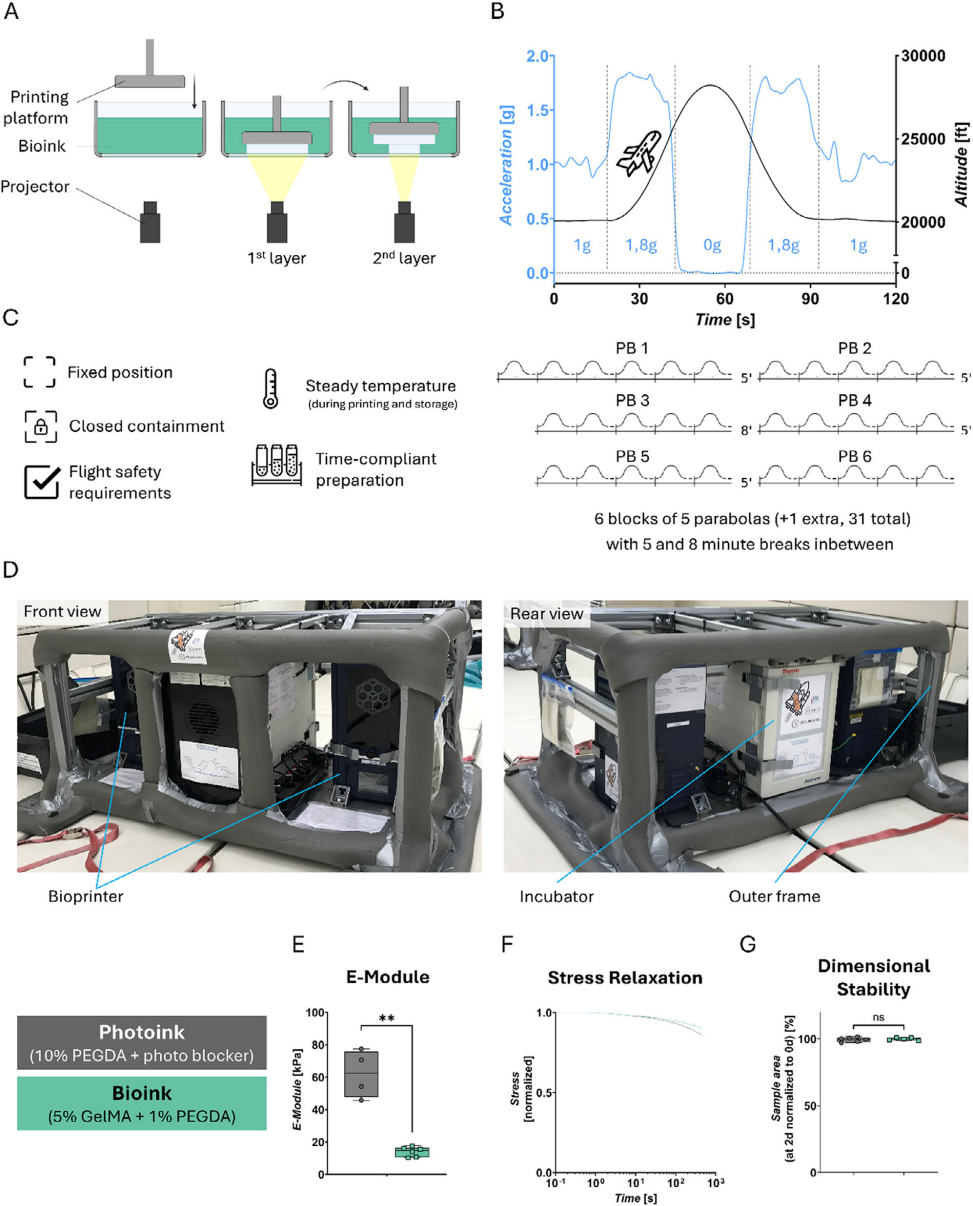
DLP in different gravitational phases – Set‐up: (A) Basic principle of DLP 3D printing; the hydrogel is bioprinted in a layer‐by‐layer manner, suspended from a printing platform through UV‐induced polymerization of the bioink. (B) Parabolic flight; a plane moving on a parabola‐shaped flight path (z‐direction) undergoes phases with changing gravitational conditions ranging from 0 to 1.8 g. One flight consists of 31 parabolas in 6 blocks with 5 and 8 min breaks in between. (C) Requirements for experimental procedure and hardware. **D** Parabolic flight experiment rack. Two DLP bioprinters and an incubator mounted in a strut frame. (E‐G) Biomaterial characterization of material systems Photoink and Bioink for stiffness (E, statistical comparison was performed via welch's t‐test, Photoink *n* = 4, Bioink *n* = 6), stress relaxation (F), and dimensional stability (G), statistical comparison was performed via unpaired t‐test, Photoink *n* = 6, Bioink *n* = 5). The data in (E,G) are expressed as the mean ± SD values. *p < 0.05, **p < 0.01, ****p < 0.0001, ns no significance. Created using Biorender.com.

Given the dynamic conditions experienced in space, such as gravitational alterations, the need for more robust and reproducible bioprinting techniques is becoming evident [1, 8]. Yet, most bioprinting systems, in particular extrusion‐based, are highly susceptible to environmental disturbances, including temperature fluctuations, light exposure and accelerative perturbations [19, 20]. Especially accelerative forces, including vibrations, are critical for unsupported, additive layering of ink in a strand‐ or droplet‐wise manner [20], as they can cause displacement of deposited material, differences in object density or even collapse of support‐free structures, if not optimized for a given accelerative state. Therefore, alternating gravitational conditions can lead to different object characteristics within a single print [21, 22, 23]. In contrast, with DLP, an object is rapidly printed in a layer‐by‐layer manner [19], suspended from a printing platform by UV‐induced polymerization of the bioink (Figure 1A). During the printing process, both the attachment of the print to the printing platform and the submerged configuration of the print within the ink bath are expected to mitigate accelerative disturbances [24]. Thus, DLP appears as a promising candidate for bioprinting tolerant to accelerative disturbances. Additionally, it offers high resolution [25, 26], in multi‐material bioprinting with reduced mechanical stress on cells during fabrication [14, 27, 28, 29, 30, 31].

This study investigated the robustness of a DLP bioprinting process under challenging gravitational conditions to assess its potential for spaceflight applications, using parabolic flights as a test platform for achieving 0–1.8 g conditions. Following the successful establishment of the material systems under the constraints of the flight environment, the process was extended to evaluate the bioprinting of constructs containing living human cells, which confirmed the potential of DLP for space tissue engineering. Furthermore, the ability of DLP 3D printing to fabricate complex hydrogel architectures under MixedG_Flight_ conditions was assessed to determine the limits of this technology in a dynamic gravitational environment.

## 2 Results and Discussion

### 2.1 Experimental Setup for DLP 3D Printing Under Varying Gravitational Conditions

As a test environment for accelerative disturbances, parabolic flight conditions were chosen, with gravitational conditions repeatedly varying between 0, 1, and 1.8 g (Figure 1B). Experiments were conducted under these gravitational conditions (MixedG_Flight_) and compared to 1 g control conditions on the ground and in‐flight (1G_Ground_ and 1G_Flight_, respectively). The parabolic flight environment required the design of a specific experimental setup. This design included fixed positioning of equipment, closed containment of fluids, temperature maintenance, and time‐efficient preparation, all while complying with strict flight safety regulations (Figure 1C), leading to the development of a DLP bioprinting system with a closed cartridge configuration. The system was integrated into a specialized experimental flight rack (Figure 1D), which housed two DLP bioprinters and an incubator for sample storage, with all components enclosed within a strut frame to ensure stability during flight.

To evaluate the potential of DLP 3D printing, we employed a systematic approach using two distinct material systems. Both material systems are well‐established and widely‐used [32, 33, 34] – a Photoink (PEGDA‐based) and a Bioink (GelMA‐based) – were chosen to maximize the applicability of the results for the bioprinting community. Moreover, Bioink was used in bioprinting experiments to prevent cell accumulation through sedimentation due to its high viscosity [14, 35].

Conceptually, our approach consisted of three steps: 1) establishing a technological proof‐of‐concept for DLP hydrogel printing under varying gravitational conditions using a Photoink benchmark model, 2) transitioning to cell‐compatible Bioink and assessing the influence of gravitational changes, and 3) bioprinting multilayered, cone‐shaped constructs with embedded human cells to evaluate structural integrity, cell accumulation, and viability.

Photoink (10% polyethylene glycol diacrylate, PEGDA) was used for an optimized resolution [7, 36, 37], while minimizing cytotoxic effects [15, 38], and printing time [39, 40]. Given the cytotoxicity of the PEGDA‐based Photoink, a cell‐compatible Bioink formulation was subsequently utilized. To reduce excitation time while preserving cell viability, the Bioink consisted of gelatin methacryloyl (GelMA, 5%) with added PEGDA (1%) [7, 41]. Before flight, printing with both materials was tested (Figure 1E–G). The elastic modulus of Photoink‐derived hydrogels, measured immediately post‐printing, was approximately four times higher (mean = ∼62 kPa) than in Bioink‐derived hydrogels (mean = ∼14 kPa), suggesting an increased challenge in maintaining geometric fidelity when printing with the mechanically softer Bioink. Both materials exhibited predominantly elastic material behavior properties and showed no swelling in isotonic buffer within two days of printing.

Earlier studies showed a gravity‐dependent increase in inhomogeneities of photopolymerized hydrogels compared to fabrication in real microgravity [42, 43], as well as in simulated microgravity [44]. A difference in hydrogel homogeneity in each layer depending on the gravitational phase present during printing must therefore be presumed for our printed hydrogels, but an in‐depth analysis was not within the scope of this study.

### 2.2 Operational Reliability of DLP Hydrogel Printing Under Varying Gravitational Conditions

**FIGURE 2:**
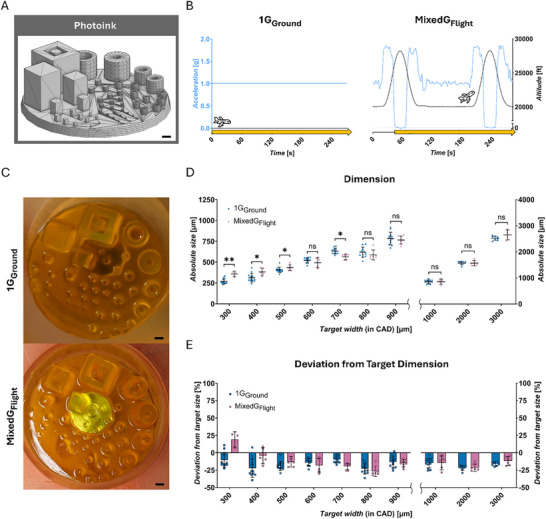
Photoink ‐ technological proof‐of‐concept of DLP 3D hydrogel printing in challenging gravitational conditions. (A) Target objects: Benchmark design (CAD). (B) Gravitational conditions tested: 1G_Ground_ and MixedG_Flight_; schematic drawing of altitude [ft] and the respective acceleration in z‐direction [g], printing process (yellow arrow). (C) Representative image of the printed hydrogel correspondent to tested condition from (B). (D) Absolute measured sizes [µm] of printed hydrogels; target width (from CAD): 300 to 900 µm (left) and 1000 to 3000 µm (right). (E) Deviation from target dimension [%]; target width (from CAD): 300 to 900 µm (left) and 1000 to 3000 µm (right). Statistical comparisons were performed via Mann‐Whitney test (300, 400, 700, 800 µm) and unpaired t‐test (500, 600, 900–3000 µm). Number of replicates: 300 µm (MixedG_Flight_ *n* = 3, 1G_Ground_ *n* = 12); 400, 1000 µm (MixedG_Flight_ *n* = 5, 1G_Ground_ *n* = 15); 500‐800 µm (MixedG_Flight_ *n* = 6, 1G_Ground_ *n* = 15); 900 µm (MixedG_Flight_ *n* = 9, 1G_Ground_ *n* = 15); 2000 µm (MixedG_Flight_ *n* = 5, 1G_Ground_ *n* = 10); 3000 µm (MixedG_Flight_ *n* = 6, 1G_Ground_ *n* = 10). The data in (D,E) are expressed as the mean ± SD values. *p < 0.05, **p < 0.01, ****p < 0.0001, ns no significance. Scale bars = 1000 µm.

To assess the functionality of DLP hydrogel printing under varying gravitational conditions, we first used the Photoink material to print a benchmark CAD model consisting of cubic and cylindrical structures of varying sizes across all conditions (Figure 2A; Figure S1A). Samples printed in MixedG_Flight_ condition were compared to those printed in 1G_Ground_ condition (Figure 2B). Printing performance was evaluated by quantification of the dimension of a cylindrical geometry in a printed sample.

Initially, the printable feature dimension range was investigated, extending from 50 to 3000 µm target width. Hydrogel constructs were successfully printed above 300 µm target width, with consistent appearance and measurable dimensions, indicating effective material solidification. However, below 300 µm target width, no hydrogel formation could be observed (Figure S2A). The dimensions of the printed constructs could be modulated in a controlled manner, allowing variation in feature size and overall geometry (Figure 2C). Next, the influence of gravitational changes and in‐flight environment on hydrogel dimensions was assessed across both conditions. Above a target width of 700 µm, no significant differences between MixedG_Flight_ and 1G_Ground_ conditions were observed. In contrast, at 500 µm target width and below, hydrogels printed in 1G_Ground_ were significantly smaller than those printed in‐flight (Figure 2D). Notably, the discrepancy in hydrogel dimensions observed between gravitational conditions became more pronounced at smaller target widths, with the minimum printable hydrogel width determined to be 357.6 ± 33.06 µm (*n* = 3) in MixedG_Flight_ and 269.1 ± 32.57 µm (*n* = 12) in 1G_Ground_. This phenomenon at finer feature scales may be attributed to either the altered gravitational profile during parabolic flight (Figure 2B) or to other factors inherent to the flight environment (e.g., vibrations).

Together, these findings demonstrate the feasibility of DLP hydrogel printing under varying gravitational conditions, confirming our initial expectation of the technologies’ tolerance against accelerative disturbances and establishing a foundation for subsequent experiments toward bioprinting.

### 2.3 Printing Resolution of DLP Hydrogels Under Varying Gravitational Conditions

**FIGURE 3:**
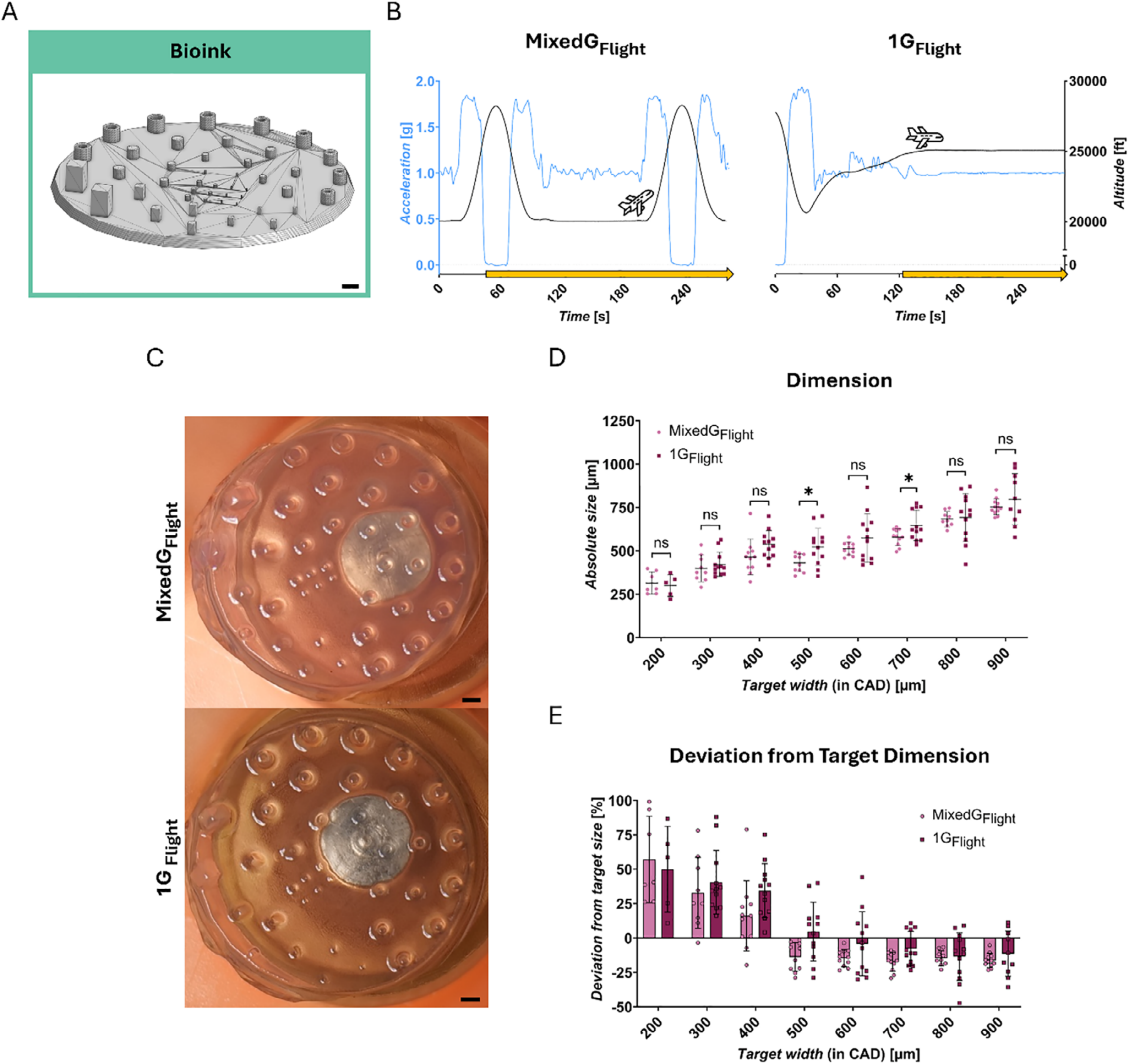
Bioink ‐ preparation for bioprinting: technological proof‐of‐concept of DLP 3D hydrogel printing in challenging gravitational conditions. (A) Target objects: adapted Benchmark design (CAD). (B) Gravitational conditions tested: MixedG_Flight_ and 1G_Flight_; schematic drawing of altitude [ft] and the respective acceleration in z‐direction [g], printing process (yellow arrow). C Representative image of the printed hydrogel correspondent to tested condition from (B). (D) Absolute measured size [µm] of printed hydrogels; target width (from CAD): 200 to 900 µm. (E) Deviation from target dimension [%]; target width (from CAD): 200 to 900 µm. Statistical comparisons were performed via unpaired t‐test (200, 400, 700 µm), Mann‐Whitney test (300 µm) and welch's t‐test (500, 600, 800, 900 µm). Number of replicates: 200 µm (MixedG_Flight_ *n* = 5, 1G_Flight_ *n* = 7); 300, 800 µm (MixedG_Flight_ *n* = 9, 1G_Flight_ *n* = 12); 400, 600, 700 µm (MixedG_Flight_ *n* = 11, 1G_Flight_ *n* = 12); 500 µm (MixedG_Flight_ *n* = 10, 1G_Flight_ *n* = 12); 900 µm (MixedG_Flight_ *n* = 12, 1G_Flight_ *n* = 10). The data in (D,E) are expressed as the mean ± SD values. *p < 0.05, **p < 0.01, ****p < 0.0001, ns no significance. Scale bars = 1000 µm.

To investigate the potential of using DLP under challenging gravitational conditions for bioprinting, we tested the previously developed printing setup using a cell‐ready Bioink. We first focused on applying cell‐free Bioink to exclude potentially interfering effects associated with the presence of viable cells in the printing system. Bioink formulations typically do not include a photo blocker, and the molecular structure of GelMA facilitates a higher light scattering [45], necessitating adjustments in print geometry to prevent overcuring. We therefore had to limit light exposure per layer to facilitate the fabrication of fine structures, which we exemplified using a cylindrical geometry with submillimeter‐scale features (Figure 3A; Figure S1B). To distinguish between the effects of the flight environment and gravitational changes, we conducted a direct comparison between hydrogel printing in MixedG_Flight_ and in‐flight 1 g control conditions (1G_Flight_, Figure 3B).

Initially, the range of printable feature dimensions was investigated, spanning target width from 50 to 1000 µm. Hydrogel constructs were successfully printed above 200 µm target width, with consistent appearance and measurable dimensions, indicating effective material solidification. However, below 200 µm target width, no hydrogel formation could be observed (Figure S2B). The dimensions of the printed constructs could be modulated in a controlled manner, allowing variation in feature size and overall geometry (Figure 3C). Next, the influence of gravitational changes on hydrogel dimensions was assessed across both conditions. Statistically significant differences were observed only at discrete feature sizes between MixedG_Flight_ and 1G_Flight_ conditions (Figure 3D), with the minimum printable hydrogel width determined to be 314.2 ± 62.99 µm (*n* = 7) in MixedG_Flight_ compared to 299.9 ± 62.4 µm (*n* = 5) in 1G_Flight_. These findings suggest the absence of a global condition‐dependent effect, while the slightly lower resolution observed for the Bioink compared to the Photoink prints can be attributed to increased overcuring in the absence of a photo blocker.

As the accuracy of targeted gravitational states (0 g, 1 g) can exhibit inherent fluctuations between flight days, the comparability between replicates in each condition may be affected (Figure S4A,B). Remarkably, no flight‐day‐specific trend in dimensional differences or coefficient of variation could be observed (Figure S3A), indicating the robustness of the printing system. Bioink‐hydrogels exhibited an elastic modulus four times lower than that of Photoink‐hydrogels, reflecting their intended cell culture application. Notably, we could not identify any significant differences in maximum resolution between both materials used (Figure S2C), underlining the versatility of DLP bioprinting regarding the use of different material and ink systems.

In summary, the absence of a globally significant difference between MixedG_Flight_ and 1G_Flight_ samples suggests that DLP hydrogel printing resolution is independent of gravitational variation and the choice of the material system. These results provide a robust foundation for advancing to the next step: in‐flight bioprinting with cell‐laden Bioinks.

### 2.4 Structural Integrity of DLP Cell‐Containing Hydrogels Under Varying Gravitational Conditions

**FIGURE 4:**
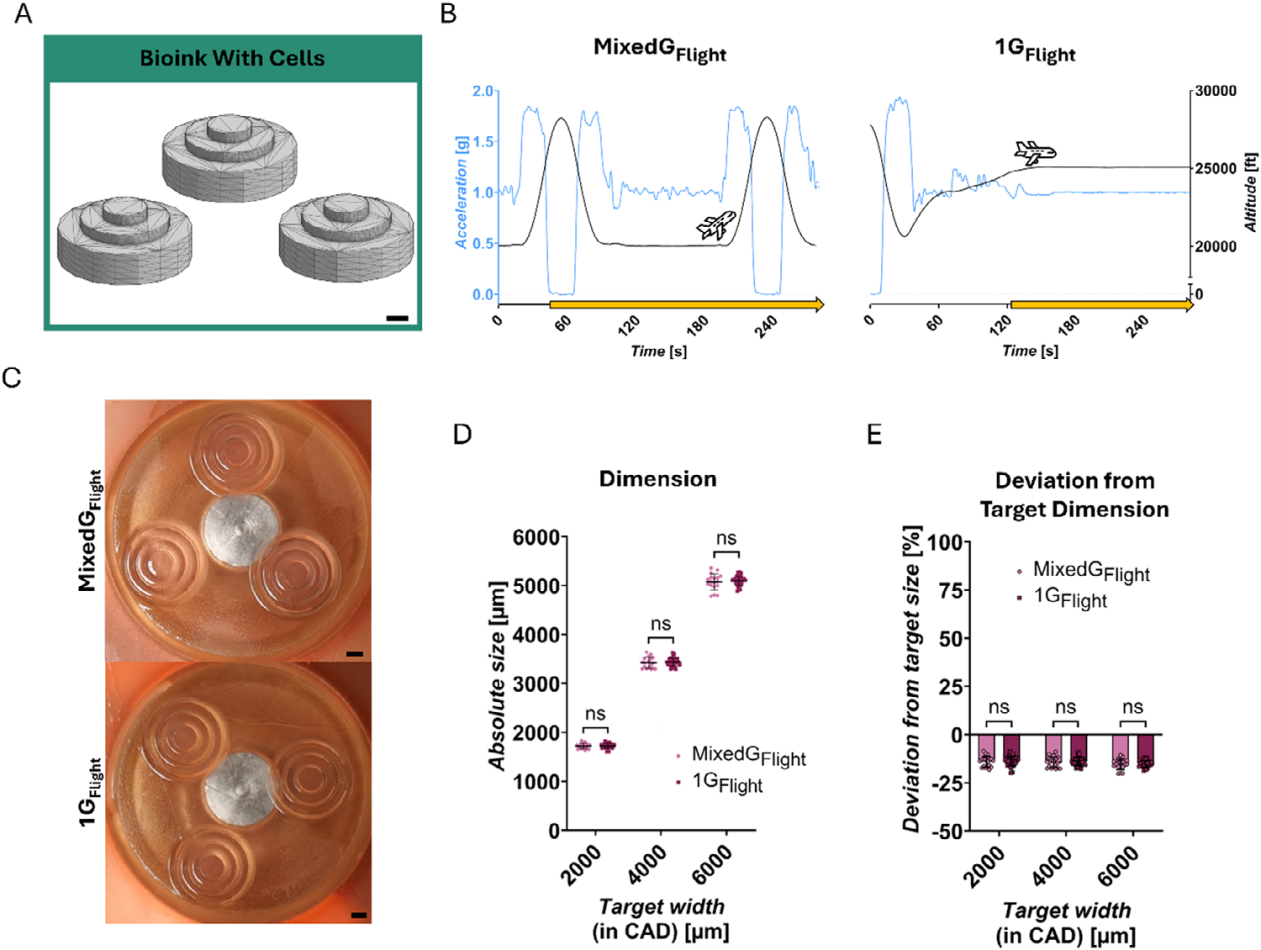
Bioink with Cells ‐ technological proof‐of‐concept of DLP 3D bioprinting in challenging gravitational conditions. (A) Target objects: multilayered, cone‐shaped design (CAD). (B) Gravitational conditions tested: MixedG_Flight_ and 1G_Flight_; schematic drawing of altitude [ft] and the respective acceleration in z‐direction [g], printing process (yellow arrow). (C) Representative image of the printed hydrogel correspondent to tested condition from (B). (D) Absolute measured size [µm] of bioprints; target width (from CAD): 2000, 4000, 6000 µm. (E) Deviation from target dimension [%]; target width (from CAD): 2000, 4000, 6000 µm. Statistical comparisons were performed via unpaired t‐test (2000, 4000 µm) and welch's t‐test (6000 µm). Number of replicates: MixedG_Flight_ *n* = 18, 1G_Flight_ *n* = 28. The data in (D,E) are expressed as the mean ± SD values. *p < 0.05, **p < 0.01, ****p < 0.0001, ns no significance. Scale bars = 1000 µm.

In light‐based methods, like DLP, the presence of cells in a bioink can influence the polymerization dynamics by causing light scattering [7, 46, 47]. To evaluate the suitability of the established material system with viable human cells, we next investigated DLP in‐flight 3D‐bioprinting performance with cell‐laden Bioink by quantifying the dimensions of cylindrical geometries (Figure 4A; Figure S1C) in a printed sample. Similar to the previous Bioink‐only experiment, we conducted a direct comparison between hydrogels printed in MixedG_Flight_ and under in‐flight 1 g control conditions (1G_Flight_, Figure 4B). As skin deterioration represents a critical concern during space travel [48], we incorporated either fibroblasts or keratinocytes – two key cell types involved in skin regeneration [9] – into the Bioink, with the long‐term objective of enabling future wound patch bioprinting in space.

In line with the previous experiment using cell‐free Bioink, cell‐laden hydrogel constructs were printed with feature dimensions of 2000, 4000, and 6000 µm, exhibiting consistent appearance and dimensions, indicative of effective material solidification. Moreover, the dimensions of the printed constructs were modulated in a controlled manner, allowing variations in feature size, despite the presence of cells (Figure S2D). Furthermore, no significant differences in hydrogel dimension were observed across the conditions (Figure 4D), reinforcing the reproducibility of DLP bioprinting under gravitational variation. Additional replicates further confirmed that gravitational changes did not affect resolution, and that cell concentration, cell type and flight day had no discernible impact on the final hydrogel dimension, suggesting polymerization remained consistent (Figure S3C–G).

These results demonstrate the dimensional consistency and robustness of the DLP bioprinting process with different cell types under accelerative disturbances in challenging gravitational conditions.

### 2.5 Deviation from Target Dimensions and Resolution Limits of In‐Flight DLP Bioprinting

Target dimensions defined in CAD models can differ from the absolute dimensions of printed structures, which is a well‐documented discrepancy observed in DLP across various materials [49, 50, 51, 52, 53]. The discrepancies arise during the polymerization process due to the replacement of van der Waals interactions between monomers with covalent bonds [54, 55, 56]. In this study, all DLP‐printed hydrogels exhibited a consistent negative deviation (∼15%) for target width above 500 µm (Figures 2E and 3E). Interestingly, for smaller feature sizes, this deviation decreased, eventually shifting to a positive value – an effect observed for both material systems exclusively under in‐flight conditions. In contrast, samples printed in 1G_Ground_ conditions using Photoink maintained a consistent negative deviation across all sizes. Thus, the shift toward overcuring observed in‐flight is likely attributable to general disturbances inherent to the flight environment. Such disturbances may enhance local mixing of the ink at the microscale during polymerization, contributing to excessive light scattering and curing beyond the intended boundaries. This phenomenon manifested itself as blurred or irregularly expanded edges. While negligible in larger features, this effect became increasingly pronounced at smaller scales, ultimately limiting the maximal achievable resolution under in‐flight conditions. Due to the various challenges of a first study on establishing DLP bioprinting in the complex conditions of parabolic flight, and the highly limited scope of experiments possible in this expensive test environment, our focus was not on optimizing the printing resolution to the highest performances reported in literature [45], but on overall feasibility of DLP bioprinting under challenging gravitational conditions. An LCD‐DLP system based on a commercially available platform was chosen due to its simpler and more compact optical configuration, despite its lower feature resolution compared to conventional DLP [57]. In addition, each hardware‐material combination has an inherent resolution limit dictated by the pixel resolution of the projection source and the inks’ polymerization kinetics [31, 45]. In Photoink, the photo blocker successfully mitigated overcuring effects, and features below the resolution threshold failed to polymerize, thereby establishing a definitive lower limit for the achievable feature size. In contrast, Bioinks – lacking a photo blocker – exhibited a progressive increase in deviation below 700 µm, with overcuring becoming more pronounced at smaller feature sizes. Despite this, the minimum printable hydrogel width was comparable to that obtained with Photoink, demonstrating that controlled overcuring can be leveraged to achieve finer structures beyond the materials’ inherent resolution threshold. Interestingly, 1G_Flight_ samples exhibited a shift from negative to positive deviation at larger target widths compared to samples printed in MixedG_Flight_, potentially due to small but persistent accelerations in the z‐direction during the 1G_Flight_ phase (Figure S4A,B). These fluctuations likely enhanced the previously described overcuring effect along the edges and reduced the achievable resolution in flight. Notably, the presence of cells in Bioinks had no measurable impact on dimensional deviation, with all prints maintaining a consistent negative deviation from the target dimensions (Figure 4E; Figure S3E,G).

In general, the dimensional deviation varied with the material system used and the dimensional deviations from target dimensions seem to depend on outside disturbances of the in‐flight environment while being independent from gravitational changes.

Hydrogels printed in microgravity‐only conditions using Xolography showed a higher achievable feature size compared to the results achieved in this study using DLP in mixedG_Flight_ conditions [58]. Nevertheless, DLP, in contrast to Xolography [59], is well‐established for bioprinting with high feature resolution [25, 26], even in gravitationally challenging environments, as confirmed by our data.

In summary, we observed a similar deviation from target dimensions for all tested gravitational conditions and identified the systems’ resolution limits depending on the flight environment, underlining the suitability of the DLP bioprinting technology for gravitationally challenging environments.

### 2.6 Bioprinting Under Varying Gravitational Conditions ‐ Cell Viability

Maintaining a high cell viability remains a key challenge for printing cells in biomaterials [6, 7]. DLP bioprinting in general provides a high cell viability [7] and therefore is assumed to introduce rather low stresses on cells. Altered gravitational conditions represent a different stressor for cells. Here, we concentrate on the short‐term effect of the combination of the bioprinting process and the altered gravity.

**FIGURE 5:**
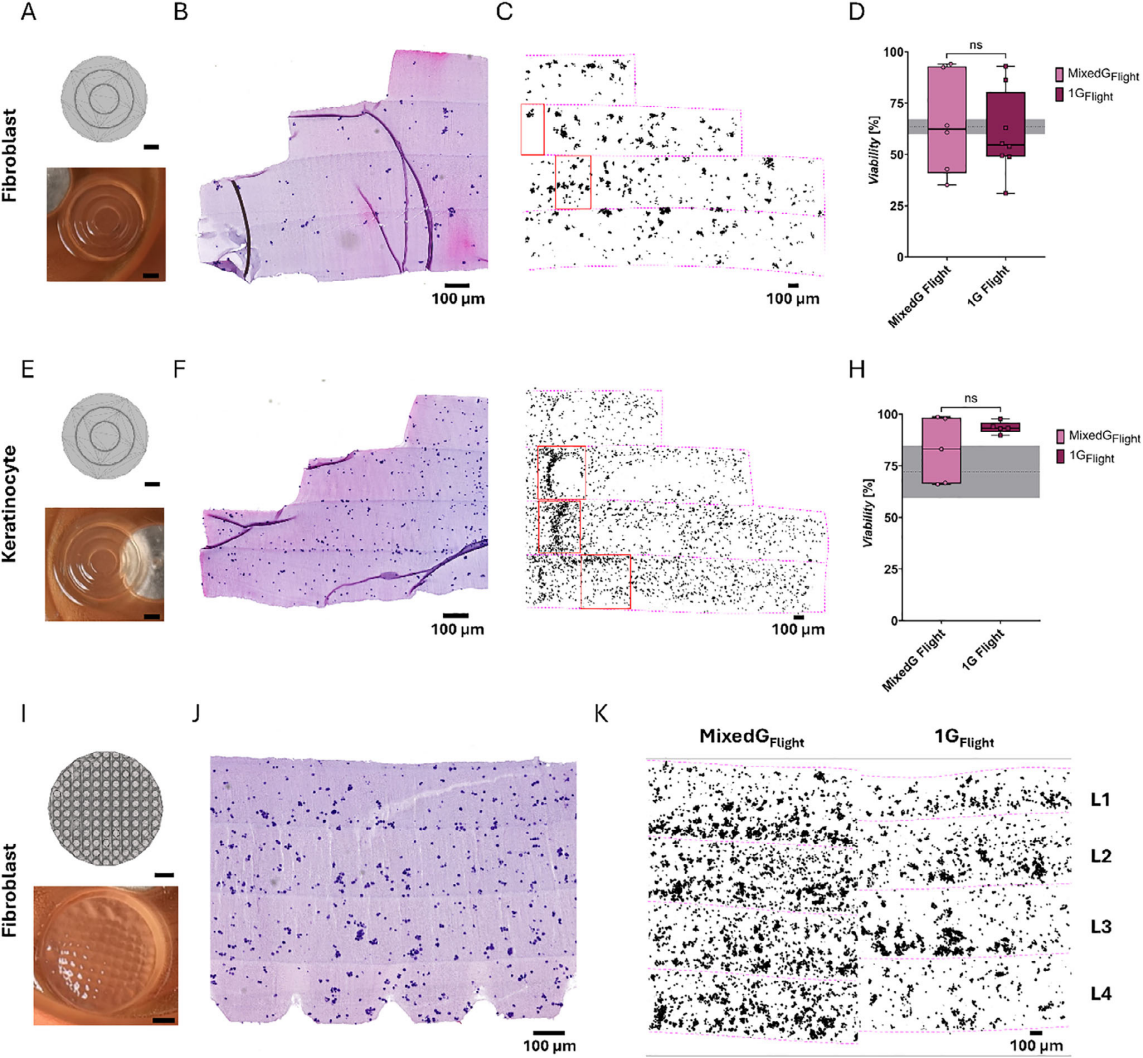
Bioink with Cells – cell viability and cell accumulation of bioprinted fibroblast and keratinocyte cells. (A,E,I) Target object (CAD design, top) and image of in MixedG_Flight_ bioprinted hydrogel (bottom); scale bar = 1000 µm. (B,F,J) Respective H&E‐stained 5 µm section with 4 mio. cells per ml (B,F) and 12 mio. cells per ml (J). (C,G) Respective cell accumulation over 105 µm thickness, with exemplary regions of cell accumulation highlighted in red; scale bar = 100 µm. (K) Cell accumulation of fibroblasts with 12 mio. cells per ml, bioprinted in MixedG_Flight_ (left) and 1G_Flight_ (right) over 105 µm thickness in 4 printed layers (L1‐L4). (D,H) Cell viability of samples bioprinted in MixedG_Flight_ and 1G_Flight_ throughout the whole campaign of the respective cell type with expectable cell viability after thawing (dotted line, SD in gray) [61]. Results for bioprinted fibroblast (A‐D) and keratinocyte (E‐H) cells. Results for fibroblast cells bioprinted with complex surface structures and high cell concentration (I‐K). Statistical comparisons were performed via Mann‐Whitney test (Fibroblast) and welch's t‐test (Keratinocyte). Number of replicates: Fibroblast (MixedG_Flight_ *n* = 6, 1G_Flight_ *n* = 8); Keratinocyte (MixedG_Flight_ *n* = 5, 1G_Flight_ *n* = 5). The data in (D,H) are expressed as 25th to 75th percentiles (box) with the median and whiskers indicating the minimum and maximum value. *p < 0.05, **p < 0.01, ****p < 0.0001, ns no significance. (A,E,I) Scale bars = 1000 µm; (B,C,F,G,J,K) scale bars = 100 µm.

Cell viability in hydrogels printed during MixedG_Flight_ and 1G_Flight_ was assessed using TUNEL staining (Figure S5A,D,G). For fibroblasts, we observed a viability of 64.88% ± 24.5% (*n* = 6) for MixedG_Flight_ and of 60.05% ± 20.4% (*n* = 8) for 1G_Flight_. For keratinocytes, we observed a viability of 82.5% ± 15.96% (*n* = 5) for MixedG_Flight_ and of 93.57% ± 2.851% (*n* = 5) for 1G_Flight_. Strikingly, no significant differences in cell viability were observed between both conditions for either cell type (Figure 5D,H). The observed cell viabilities are in line with expectations, as the cells were stored and transported in a frozen state on dry ice [60] and mixed into the ink immediately after thawing, a procedure that has been reported to reduce initial viability [61]. A potential factor influencing cell viability could be the printing time point within a flight, as cells printed during a late parabola are exposed for longer to potentially toxic unreacted photo initiator than cells printed earlier. In addition, the previously mentioned influence of an individual flight day could also contribute to variations in cell viability. Notably, both cell types were not influenced differently by either the individual flight day or the printing time point (Figure S5). Temperatures within the cabin environment, printers, and incubator remained stable throughout each flight, ensuring consistent thermal conditions during printing (Figure S4C–F). The printing process is independent of the environmental humidity due to the closed system. The previously described factors, including the handling by individual team members, did not affect cell viability. Despite not statistically significant, a trend toward a higher variability in mixedG_Flight_ appeared to be present compared to 1G_Flight_, especially in Keratinocytes compared to Fibroblasts.

In summary, these findings are remarkable as we could show that DLP bioprinting allowed us to bioprint viable human cells under challenging gravitational conditions with no significant negative effects on their viability.

### 2.7 Bioprinting Under Varying Gravitational Conditions ‐ Cell Accumulation

Since precise and controlled placement of cells plays a key role in the generation of biologically viable constructs suitable for tissue regeneration [11, 18], it is crucial to evaluate cell accumulation within hydrogels to determine whether varying gravitational conditions influence sedimentation behavior. Given the absence of gravitational forces in gravity conditions below 1 g, reduced or absent sedimentation can be expected, whereas gravity conditions above 1 g are anticipated to enhance sedimentation and accumulation effects. A parabolic flight features not only phases of microgravity, but also hypergravity, mitigating cell accumulation and sedimentation. Therefore, Bioink was used in bioprinting experiments, as these GelMA/PEGDA composites are characterized by relatively high viscosity [41], which inherently supports a more uniform cell distribution by reducing cell sedimentation during printing. This effect is attributed to the slower gravitational settling of cells in viscous environments [14]. Importantly, variations in cell concentration do not substantially affect the viscosity of the ink, ensuring consistent rheological behavior across different formulations [35].

The cell accumulation was analyzed in simple cylindrical geometries (Figure 5A–C,E‐G), as well as in a more complex architecture (Figure 5I–K), mimicking the rete ridge structure of primary skin [62]. In 1G_Flight_ conditions, cell sedimentation was moderate across all printed layers, even at higher cell concentrations, resulting in a largely homogeneous distribution (Figure 5K right, Figure S6B, right). However, in MixedG_Flight_ conditions, a higher cell aggregation compared to the normal gravity condition, could be detected (Figure S7), presumably due to sedimentation. More cells were observed in the first and second printed layers, alongside localized clusters of cell accumulation (Figure 5C,G; Figure S6A,B,C left; representative regions highlighted in red). These observations could be confirmed for all tested cell concentrations. To further mitigate sedimentation, the ink was thoroughly mixed before cartridge insertion into the printer, and printing commenced during the microgravity phase following a brief pre‐print mixing step. Nevertheless, the results indicate that this approach, together with the choice of ink, was insufficient to prevent cell accumulation. The observed heterogeneity suggests insufficient Bioink homogenization prior to printing, as well as during subsequent layer deposition, resulting in progressive sedimentation toward the lower regions of the constructs. This is further supported by inconsistencies in layer‐specific cell concentration. Notably, in one fibroblast sample, the second layer of the 2 million cells per ml condition contained a higher number of cells than the corresponding layer in the 4 million cells per ml condition (Figure S6A, left), suggesting randomness in clustering.

Additionally, cell type‐specific differences in aggregation were noted. Fibroblasts seem to exhibit increased clustering under both conditions, whereas keratinocytes are predominantly aggregated in 1G_Flight_ (Figure S6, blue highlights; Figure S7). The discrepancy in cell‐specific behavior is likely due to intrinsic cell behavior: fibroblasts, as stromal cells, possess active migratory and contractile properties, which may influence aggregation dynamics [63], whereas keratinocytes, as epithelial cells, primarily adhere to the keratin cytoskeleton [64].

These findings indicate that alternating gravitational phases during parabolic flights contribute to increased cell accumulation due to sedimentation, and at the same time, may inhibit excessive aggregation.

### 2.8 Potential for Fabrication of Complex 3D Architectures in Space

**FIGURE 6:**
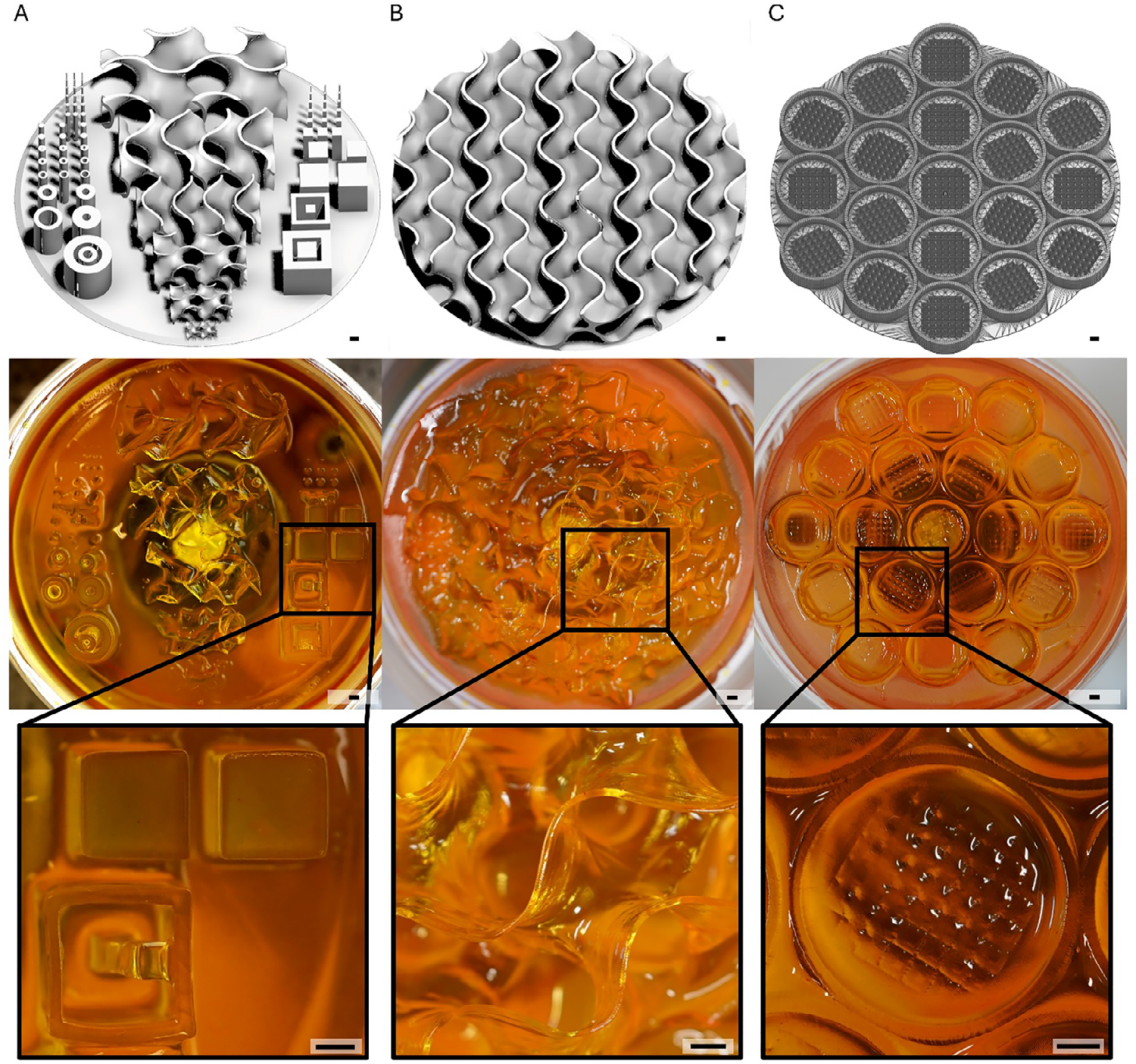
Generation of complex hydrogel architectures using DLP hydrogel printing in challenging gravitational conditions with Photoink. (A) Extended benchmark model with cubical, spherical, and gyroid structures of varying size. (B) Gyroid structure. (C) Microwell array. Target objects (CAD designs) (top), exemplary image of the correspondent printed hydrogel (middle) with zoom‐in (bottom); scale bar = 1000 µm.

Tissue functionality is closely linked to its spatial organization, as native tissues display complex 3D architectures. Common to many tissue architectures are variations of similar basic geometrical features like spherical, cubic or tube‐like shapes [6, 7]. One example are vascular structures, which are essential in tissue homeostasis and regeneration [65]. Another example are rete ridge structures in skin, which are characterized by an undulating microarchitecture that provides mechanical stability, serves as stem cell niche and promotes the epidermis’ nutrient supply [66]. To assess the applicability of our DLP system for fabricating complex 3D architectures also under conditions relevant to space flight (MixedG_Flight_), we printed three different hydrogel architectures using Photoink. The extended benchmark design (Figure 6A) showed successful printing of tube‐like structures in spherical and cubic shapes. The design also featured gyroid structures, complex geometries with advanced curvatures, of different sizes. The printed gyroids showed smooth surfaces without gaps, as well as no straight lines or flat planes. The results emphasize our printing systems’ ability to fabricate basic and complex 3D architectures. Producing tissue engineering constructs to address skin injuries is challenging due to large wound sizes and structurally complex wound environments [9]. Therefore, a larger gyroid construct (Figure 6B) was generated. Using a gyroid geometry similar to the above‐described structures, the larger model included wall thicknesses between 150 and 350 µm and extended over a width of more than 1.5 cm. Again, the printer produced smooth surfaces without gaps, as well as no straight lines or flat planes. The findings further demonstrate the printer's ability to produce continous, curved shapes of complex architectures. Biomedical research using in vitro assays in space, including studies on microgravity‐induced changes in cell behavior, drug screening, or tissue model development, is gaining importance [7, 9, 10, 11]. A representative microwell array for in vitro assays was successfully fabricated (Figure 6C), comprising 19 individual chambers. The produced array was characterized by straight edges and clearly visible individual microwells, underlining the system's potential for space‐based production of tailored in vitro experimentation.

The ability to successfully print the different complex architectures suggests the potential of the 3D printing setup for the fabrication of application‐relevant 3D constructs for future space‐based biomedical purposes.

## 3 Conclusion

This work demonstrates that DLP bioprinting represents a promising technology for space applications, with robust printing performance even in extreme conditions. We performed DLP bioprinting in parabolic flights to investigate the impact of accelerative disturbances from fluctuating gravitational conditions, which may disturb a biofabrication process during space missions. Printed hydrogel constructs retained dimensional fidelity, even in complex architectures, and supported high cell viability across various gravitational conditions. These included dynamically changing gravitation, ranging from microgravity to hypergravity during flight (0–1.8 g), as well as constant gravity during flight (1 g) and on the ground (1 g). Our data show that the viability of encapsulated cells (keratinocytes, fibroblasts) was consistently maintained across all tested conditions, highlighting robust bioprinting performance. Considering the known advantages of DLP bioprinting for tissue engineering, such as controllable, non‐invasive cell placement into high‐resolution 3D constructs, our findings position DLP as a powerful tool for next‐generation regenerative treatment strategies also in space.

Limitations of the study include the limited opportunity to optimize the system due to the finite number of parabolic flight runs. Moreover, the primary effect of accelerative forces on printing performance was observed in the accumulation of cells within printed hydrogels, underlining the need to develop hardware systems supporting enhanced autonomous mixing. Possible alterations in the hardware set‐up could be an optimization of the cartridges for an inherent larger mixing effect or the adjustment of the printing process itself, including longer and more thorough mixing through vertical movement of the print head. Moreover, an adaptation of the ink formulation using viscosity enhancers [15, 16] attenuating cell sedimentation could be beneficial in aiming to control cell distribution in space‐compatible bioprinting. While only two ink systems were evaluated in the present study, the contrasting material properties provide valuable insights for future engineering efforts toward bioprinting in space and could help refine material formulations to enable printing of finer structures to mimic tissue architecture. Future work should focus on refining printer hardware and fabrication processes, as well as mechanistic studies of in situ phenomena, including fluid dynamics, light scattering effects, microstructural [42, 44] and mechanical properties of printed hydrogels and may benefit from extended gravitational testing aboard sounding rockets as an advanced test system with more extended phases of different gravitational environments. Addressing the identified key challenges could allow to leverage the potential of the DLP printing system for biofabrication of functional tissue constructs during space flights [40], a technology already established for terrestrial use. Further studies focusing on the impact of the space environment on activity and function of bioprinted cells will provide a deeper insight into the applicability of viable tissue constructs manufactured in space for regenerative wound care. In the future, on‐demand bioprinting may contribute to astronaut healthcare during deep space missions by enabling the treatment of life‐threatening skin burns with bioprinted patient‐specific tissue grafts—potentially supporting sustained human exploration and colonization of Mars.

## 4 Experimental Section/Methods

### 4.1 Parabolic Flight Hardware

To fulfill the safety requirements of a parabolic flight, the printing process was required to be executed inside of a closed cartridge. For this purpose, the flight rack contained two modified, commercially available stereolithographic printers (Anycubic Photon Mono SE), as well as an incubator (ThermoFisher Heratherm IMC18 Bio) for the thermally controlled storage of the cartridges. All components were tightly secured in a strut frame.

### 4.2 Parabolic Flight Setup

A single parabola consisted of 3 gravitational phases: hypergravity (∼1.8 g for ∼24 s), microgravity (∼0 g for ∼22 s), and a second hypergravity phase (∼1.8 g for ∼22 s), concluded by ∼1:45 min of normal gravity (∼1 g), totaling a duration of 3 min per parabola. A parabolic flight campaign consisted of 3 consecutive flight days with 30 + 1 parabolas executed in 6 blocks of 5 parabolas with intermissions of 5, 8, and 5 min at normal gravity. In‐flight experiments were conducted during the 33th and 42nd DLR parabolic flight campaigns. Technical issues on the first flight day of the 42nd campaign reduced the parabola count to 16, necessitating an additional flight on the second day.

### 4.3 Ink Components

Poly(ethyleneglycol)diacrylate (PEGDA) (average Mn = 700 g mol^−1^) was sourced from Sigma–Aldrich Chemie GmbH (455008), while Dulbecco's Phosphate‐Buffered Saline (D‐PBS) was obtained from Corning (21‐031‐CV). Tartrazine (Sigma–Aldrich Chemie GmbH, T0388, M = 534.36  g mol^−1^) was dissolved in ultrapure distilled water (Invitrogen, 10977‐035) to prepare a 10 mm stock solution. Gelatine methacrylate (GelMA, 10% w/w stock, DoF = 67.95%) was prepared from lyophilized material in DMEM + HEPES FBS high medium (sterile filtered, 0.45 µm). Lithium phenyl‐2,4,6‐trimethylbenzoylphosphate (LAP) was pooled from multiple sources (Cellink, VLLP00010010; Advanced Biomatrix, 5269; Sigma–Aldrich Chemie GmbH, 900889) and dissolved in ultrapure distilled water to yield a 4% w/w stock. Photoink is composed of D‐PBS containing 10% PEGDA 700, 1 mm tartrazine, and 0.5% LAP. Bioink was formulated by combining the cell‐specific medium (HaCat: DMEM + HEPES FBS high medium; BJ‐Fibro and cell‐free: MEM + HEPES FBS high medium) with 5% GelMA, 1% PEGDA 700, and 0.5% LAP. The cell‐containing inks were prepared by resuspending cells in their respective Bioink at specific densities: keratinocytes (2, 4, and 16 Mio/mL) in DMEM ink; fibroblasts (2, 4, and 12 Mio/mL) in MEM ink.

### 4.4 Cell Culture

The cell types used in this study comprised a fibroblast cell line (CRL‐2522, ATCC) and a keratinocyte cell line (300493, Cell lines Services). DMEM + HEPES FBS high medium was prepared using DMEM with GlutaMAX supplement (Gibco, 61965026) supplemented with 20% fetal bovine serum (FBS Superior, Sigma–Aldrich, S0615), 1% penicillin/streptomycin (Pen/Strep, Corning, 30‐002‐Cl), and 25 mm HEPES (Fisher Bio Reagents, BP299‐100). MEM + HEPES FBS high medium was prepared from Minimum Essential Medium (MEM, Corning, 10‐010‐CV), 20% FBS, 1 mm sodium pyruvate (Corning, 25‐000‐Cl), 1X non‐essential amino acids (NEAA, Gibco, 11140‐035), 1% Pen/Strep, and 25 mm HEPES.

### 4.5 Cell Freezing and Storage

The fibroblast cell line and the keratinocyte cell line were frozen with a concentration of 30 million cells in 1 mL of their respective freeze medium. Keratinocyte freeze medium was prepared using DMEM with GlutaMAX supplement (Gibco, 61965026) supplemented with 10% fetal bovine serum (FBS Superior, Sigma–Aldrich, S0615), 1% penicillin/streptomycin (Pen/Strep, Corning, 30‐002‐Cl), 25 mm HEPES (Fisher Bio Reagents, BP299‐100), and 5% Dimethylsulfoxide (Sigma–Aldrich, D8418‐50ML). Fibroblast freeze medium was prepared from Minimum Essential Medium (MEM, Corning, 10‐010‐CV), 20% FBS, 1 mm sodium pyruvate (Corning, 25‐000‐Cl), 1X non‐essential amino acids (NEAA, Gibco, 11140‐035), 1% Pen/Strep, 25 mm HEPES, and 5% Dimethylsulfoxide (Sigma–Aldrich, D8418‐50ML). Frozen vials were stored in liquid nitrogen. For the transport to Bordeaux and the subsequent storage until thawing frozen vials were kept on dry ice at −80°C.

### 4.6 Biomaterial Measurements

The top width of the printed hydrogels was measured directly after printing and following 2 days of incubation in DMEM + HEPES FBS high medium at 37°C (imaged using a Keyence BZ‐X800 microscope and measured using Keyence Software BZ‐X8000 Analyzer Version 1.1.2.4, width normalized to 0d time point). ECM elastic and stress‐relaxation properties were quantified by elastic modulus and stress‐relaxation time by uniaxial unconfined compression (TestBench LM1 system, BOSE) using a 250‐gram load cell (Model 31 Low, Honeywell) directly after printing. The compression step was conducted with minimal pre‐load, at 0.016 mm s^−1^, and to a maximum strain of 15% along the longitudinal axis of the cylindrical samples. At maximum compression, the stamp position was held constant to record stress relaxation. The maximum compression was limited to 15% strain of tissue sample height. The elastic modulus E was calculated from the slope of a 5% interval of the linear region of the stress‐strain curve. The testing protocol restricted the recording of stress‐relaxation times to 468 s, as it was previously shown that PEGDA, and therefore eventually also PEGDA‐mixtures, are purely elastic [67].

### 4.7 Printing During Parabolic Flight

Cells were cryopreserved after expansion (30 × 10^6^ in 1 mL per vial) and transported on dry ice from Berlin, Germany to the launch side in Bordeaux, France. On flight days, vials were thawed, centrifuged, and resuspended in their respective inks. Cartridges were filled with ink (15 or 30 mL depending on the cartridge size), ensuring no air remained in the cartridges, and stored at 37°C (first ground‐based, then in flight rack incubator). For the printing process, the cartridges were mixed through manually shifting the printing platform, then loaded into the printer. The homing procedure was performed, and the print was started (for the MixedG_Flight_ samples start on the first parabolas’ microgravity phase; for the 1G_Flight_ samples either before or after the parabola blocks or during the 8 min break). The 1G_Ground_ samples were printed on ground.

### 4.8 Post‐Flight Analysis of Printed Constructs

Directly post‐flight, cartridges were disassembled and photographically documented. Cell‐containing hydrogels were washed with D‐PBS and fixed in 4% paraformaldehyde (in PBS, Thermo Scientific Chemicals, J61899‐AK). Samples were stored at 4°C before being transported back to Berlin, and hydrogels were cut in half using a sterile scalpel.

### 4.9 Hydrogel Dimension

Hydrogel dimensions were assessed by measuring the width of a printed construct. Therefore, the images taken directly post‐flight were analyzed using ImageJ v1.54f. A width was defined by the double mean value of radii probed at 50 equally spaced positions around an object on manually defined sample edges. Samples with an out‐of‐focus image plane were excluded, as they were difficult to quantify. All absolute values (mean with SD) of hydrogel dimensions (width) and number of replicates for hydrogels printed with the respective ink under the respective conditions are available in Table S1. All Percentages (mean with SD) of hydrogel deviation from target dimension and number of replicates for hydrogels printed with the respective ink under the respective conditions are available in Table S2.

### 4.10 Cell Accumulation

To evaluate spatial cell accumulation and sedimentation, one half of each construct was stained with Draq5 (Biolegend, 424101, λ_Ex_ = 633 nm, λ_Em_ = 700/50 nm) to visualize nuclei. Confocal microscopy (Leica SP5, Leica microsystems CMS, Wetzler, Germany; stitching with LASX software: Leica Application Suite X, Version 3.7.5.24914) was used to image the stained samples 105 µm into the hydrogel, with a z‐step size of 5 µm. Cell accumulation was assessed using ImageJ v1.54f with applying the Analyze Particle Plugin to binary images of Draq5+ cell nuclei of z‐projection by maximum intensity. The total area covered with cells inside of each hydrogel was determined using the resulting ROIS, and the ratio to the total hydrogel area calculated. The foldchange between the mixedG_Flight_ and the 1G_Flight_ sample was determined as a measure of the difference in cell accumulation between the conditions.

### 4.11 Cell Viability

To assess cell viability across different printing conditions, the remaining half of each sample was embedded (Tissue‐Tek, O.C.T. Compound, Sakura, 4583) and cryosectioned (Cryotome: Leica CM3050 S). The percentage of viable cells was determined by TUNEL staining (Click‐iT plus TUNEL Assay Kit, Life Technologies, C10617, λ_Ex_ = 490 nm, λ_Em_ = 525 nm) intensity (integrated density) in Draq5+ (λ_Ex_ = 620/60 nm, λ_Em_ = 700/75 nm) cell nuclei in reference to a positive control. To establish a viability threshold, an on‐ground printed sample was analyzes using a live/dead viability assay (ReadyProbes Cell Viability Imaging Kit, Blue/Red, Life Technologies, R37610) directly after printing, and the resulting viability data were used to define the cut‐off value for distinguishing viable from non‐viable cells based on TUNEL integrated density within the same sample. The samples were microscopically imaged (Leica DM6B, Leica microsystems CMS, Wetzler, Germany; with digital camera: Leica DMC 4500, Leica microsystems (Switzerland) Ltd., Heerbruch, Switzerland), stitched with LASX software (Leica Application Suite X, Version 3.7.5.24914) and analyzed with ImageJ v1.54f.

### 4.12 Statistical Analysis

The statistical analysis and the generation of the graphs were conducted on GraphPad Prism 9.5.1. For all statistical analyzes, significance level was determined as *p < 0.05, **p < 0.001, ***p < 0.001, and ****p < 0.0001. The samples’ normality was tested with a Shapiro‐Wilk and a Kolmogorov‐Smirnov test. Subsequent statistical tests are included in the respective figure legend.

## Author Contributions

Bianca Lemke, Lutz Kloke, and Georg N. Duda conceived the research. Tobias Lam designed and developed the cartridge system, as well as the 3D CAD files “extended benchmark model”, “gyroid structure”, and “microwell array”, modified the stereolithographic 3D bioprinter, and constructed the experiment rack. Bianca Lemke and Tobias Thiele conceptualized and established the experimental set‐up. Bianca Lemke organized both flight campaigns, with Tobias Lam contributing to the organization of the first campaign. Bianca Lemke and Nicolas Göbel performed the preparation for the flight campaigns. Bianca Lemke, Tobias Thiele, and Nicolas Göbel performed the in‐flight experiments. Bianca Lemke and Lisa R. Köhn processed the post‐flight samples. Gabriela Korus performed TUNEL staining. Bianca Lemke and Lisa R. Köhn generated and Bianca Lemke curated the visualizations. Bianca Lemke performed image quantifications and data analysis. Bianca Lemke, Matthias R. Kollert, Lutz Kloke, and Georg N. Duda interpreted the data. Bianca Lemke wrote the manuscript. Matthias R. Kollert contributed to editing the original draft. All authors read and approved paper.

## Funding

German Space Agency at DLR: 50WB2034, 50WB2311; European Union: ERC‐2021‐ADG, 101054501.

## Conflicts of Interest

The authors declare no conflict of interest.

## Supporting information




**Supporting File**: advs73829‐sup‐0001‐SuppMat.docx.

## Data Availability

The data that support the findings of this study are available in the supplementary material of this article.
