# Supplementary material for: Gravity‐Tolerant In‐Flight 3D Bioprinting Enabled by Stereolithography for Space Tissue Engineering
Source: Adv Sci (Weinh). 2026 Jan 21;13(17):e20715. doi: 10.1002/advs.202520715 (PMC13042947; doi:10.1002/advs.202520715)
Supplement: Supplementary file 1 — Supporting File: advs73829‐sup‐0001‐SuppMat.docx. [file ADVS-13-e20715-s001.docx]

Supporting Information

Gravity-Tolerant In-Flight 3D Bioprinting Enabled by Stereolithography for Space Tissue Engineering

Bianca Lemke, Matthias R. Kollert, Tobias Lam, Tobias Thiele, Nicolas Göbel, Lisa R. Köhn, Gabriela Korus, Lutz Kloke, Georg N. Duda*


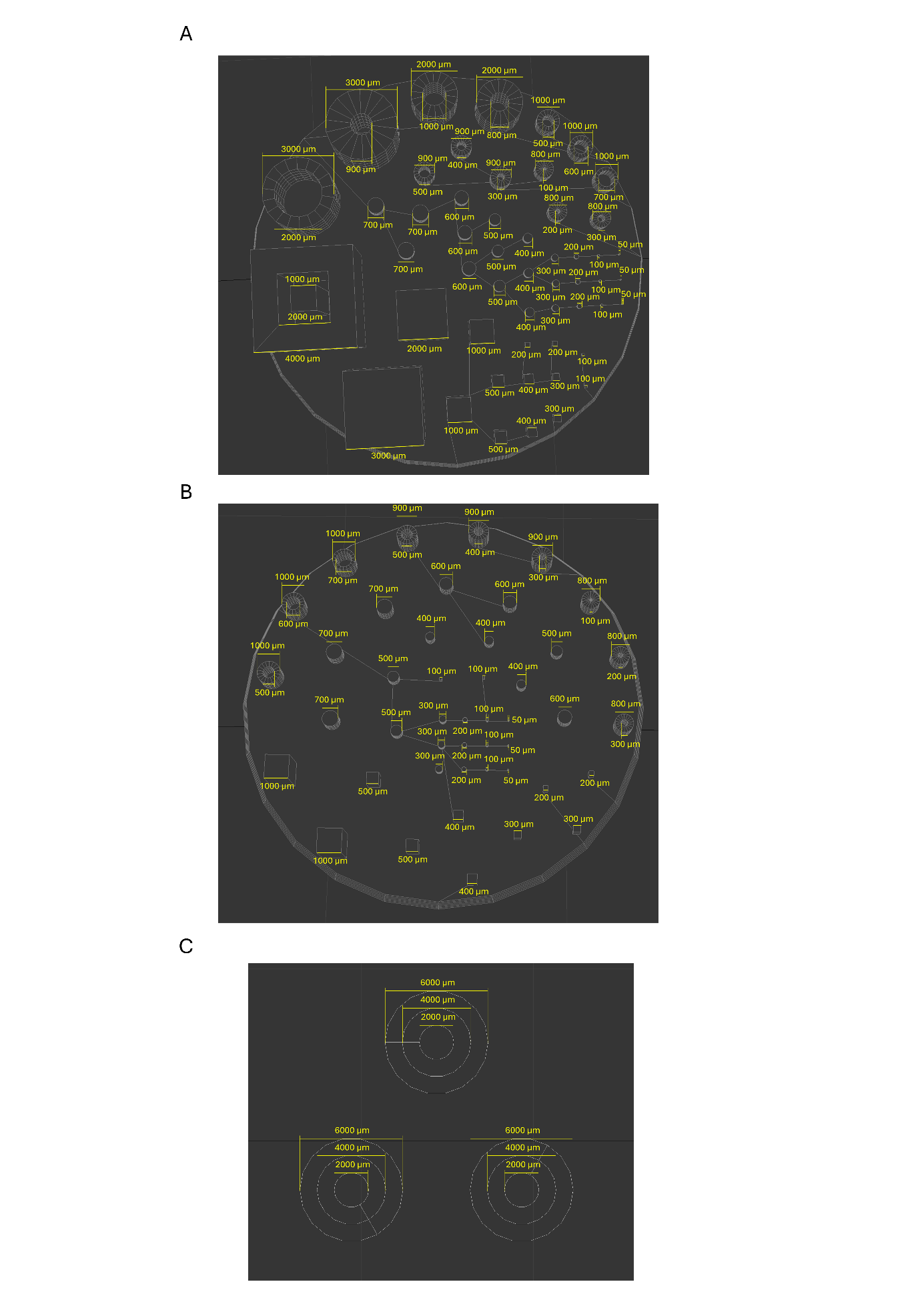


**Figure S1.** Dimensions of CAD models. **A** Benchmark model with cubic and cylindrical structures of varying sizes from 4000|3000 µm to 100|50 µm (cube|cylinder). **B** Adapted Benchmark model with cubic and cylindrical structures of varying sizes from 1000 µm to 100|50 µm (cube|cylinder). **C** Multilayered, cone-shaped design with three levels of 6000, 4000 and 2000 µm diameter (bottom to top).


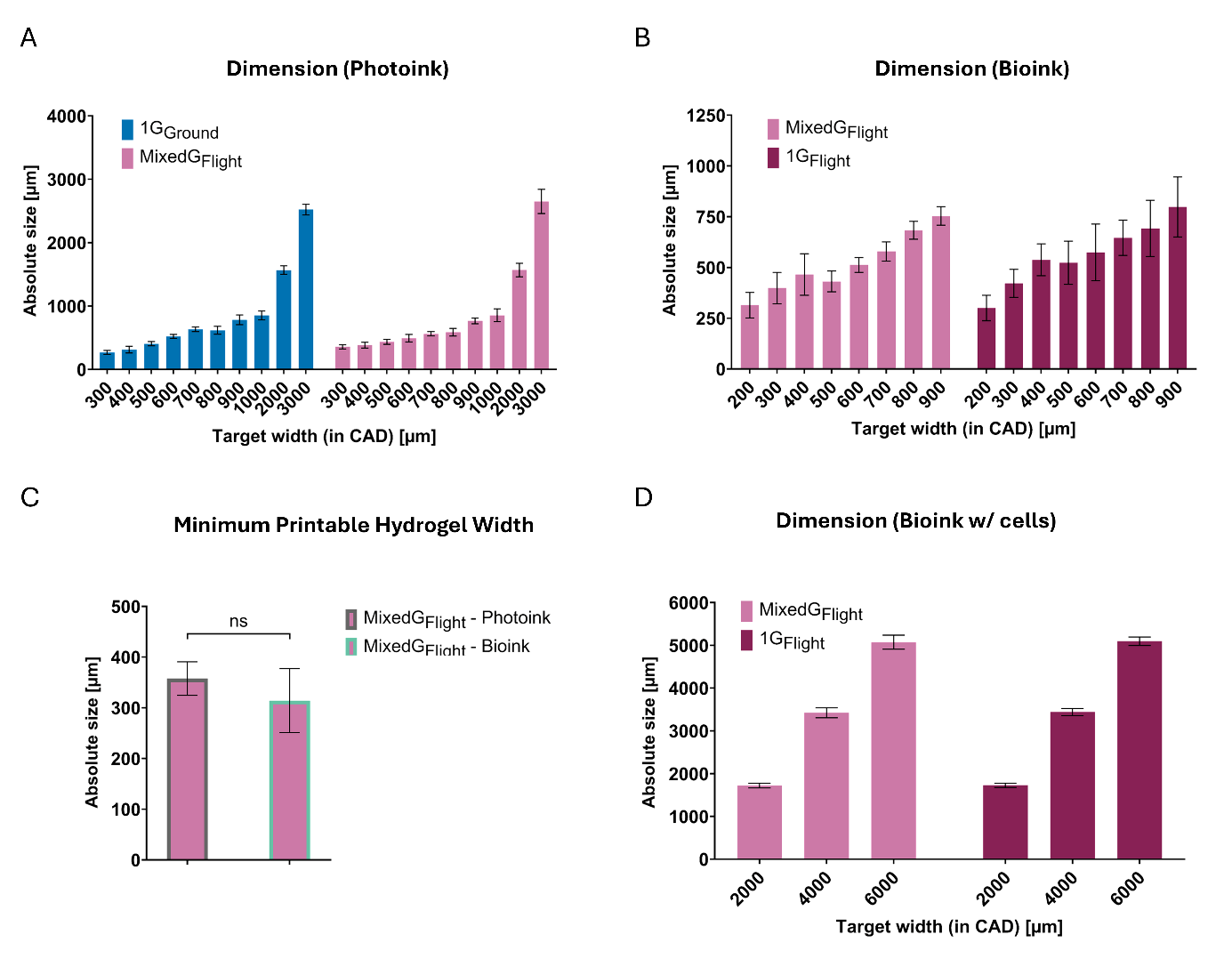


**Figure S2.** Dimensional control of printed hydrogels for all ink conditions. Hydrogel dimensions of hydrogels printed in 1G_Ground_ and MixedG_Flight_ using Photoink (**A**) and Bioink (**B**). **C** Minimum printable hydrogel width in MixedG_Flight_ for Photoink (*n* = 3) and Bioink (*n* = 7). Statistical comparison was performed via Mann-Whitney test. **D** Hydrogel dimensions of Bioink with cell hydrogels bioprinted in MixedG_Flight_ and 1G_Flight_. The data is expressed as the mean ± SD values. * p < 0.05, ** p < 0.01, **** p < 0.0001, ns no significance.


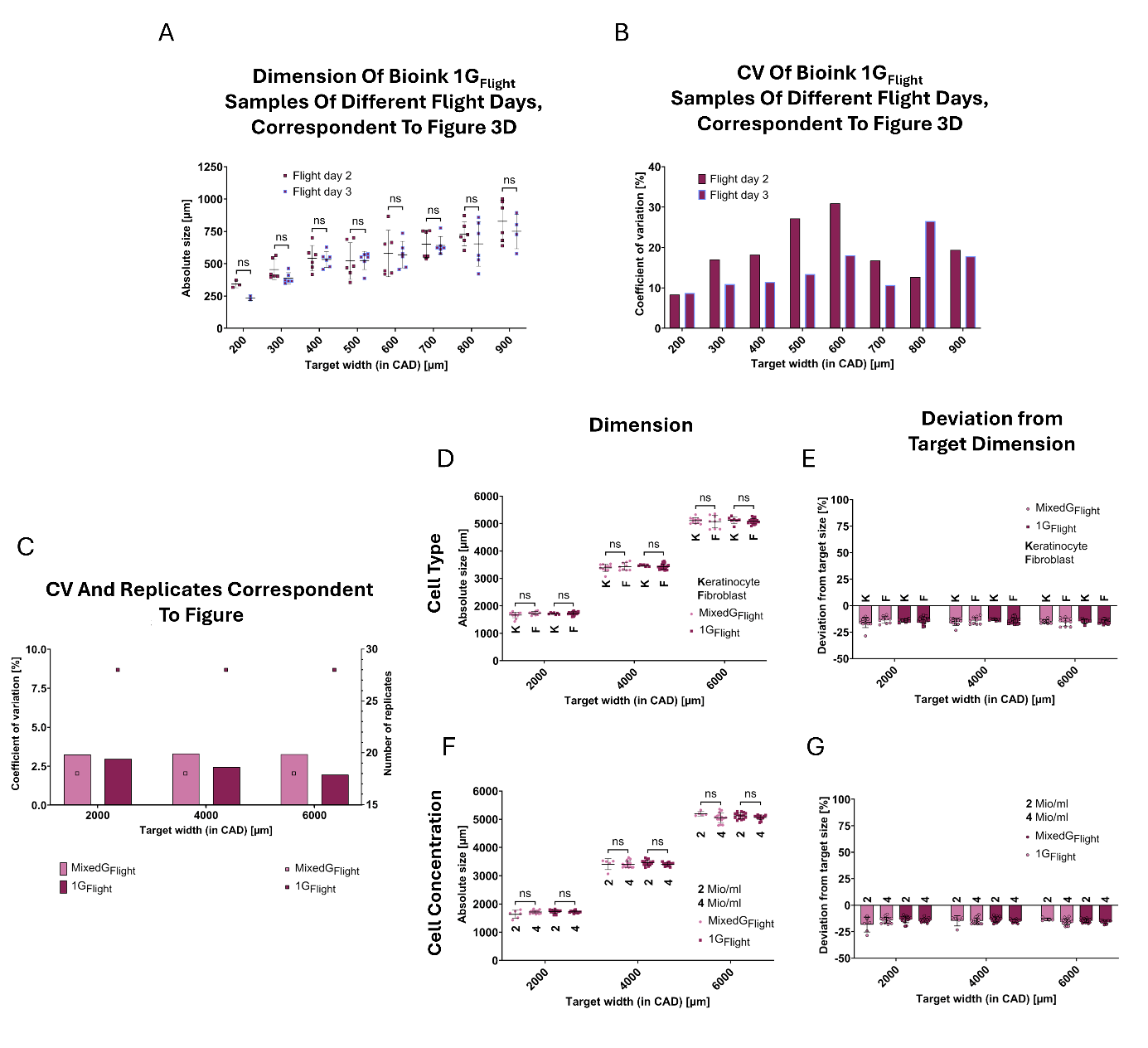


**Figure S3.** Dependency of dimensional bioprint characteristics on cell type, cell concentration and respective flight day. **A** Absolute measured size [µm] of hydrogels printed on flight days 2 and 3 correspondent to Figure 3D; target width (in CAD): 200 to 900 µm. Statistical comparisons were performed via Mann-Whitney test (200, 300, 500, 700, 900 µm) and unpaired t-test (400, 600, 800 µm). Number of replicates: 200 µm (FD2 *n* = 3, FD3 *n* = 2); 300-800 µm (FD2 *n* = 6, FD3 *n* = 6); 900 µm (FD2 *n* = 6, FD3 *n* = 4). **B** Dimensional reproducibility of printed hydrogels, coefficient of variation (CV) correspondent to Figure 3D. **C** Dimensional reproducibility of bioprinted hydrogels. Coefficient of variation (CV, bar plots) and number of replicates (individual points) correspondent to Figure 4. Influence of cell type (F – fibroblast; K – keratinocyte) on bioprint dimensions (**D**) and bioprint deviation from target dimension (**E**). Statistical comparisons were performed for MixedG_Flight_ via Mann-Whitney test (2000 µm), unpaired t-test (4000 µm) and welch’s t-test (6000 µm) and for 1G_Flight_ via unpaired t-test (2000 µm), welch’s t-test (4000 µm) and Mann-Whitney test (6000 µm). Number of replicates: MixedG_Flight_ 2000 µm (Fibroblast *n* = 9, Keratinocyte *n* = 12), 4000-6000 µm (Fibroblast *n* = 9, Keratinocyte *n* = 11); 1G_Flight_ 2000-6000 µm (Fibroblast *n* = 21, Keratinocyte *n* = 7). Correspondent for cell concentrations (2 – 2 Mio. cells per ml; 4 – 4 Mio. cells per ml; **F**,**G**). Influence of respective flight day on bioink hydrogel print dimensions. Statistical comparisons were performed for MixedG_Flight_ via unpaired t-test (2000, 6000 µm) and Mann-Whitney test (4000 µm) and for 1G_Flight_ via unpaired t-test (2000, 4000 µm) and Mann-Whitney test (6000 µm). Number of replicates: MixedG_Flight_ 2000 µm (2 Mio/ml *n* = 6, 4 Mio/ml *n* = 15), 4000-6000 µm (2 Mio/ml *n* = 5, 4 Mio/ml n = 15); 1G_Flight_ 2000-6000 µm (2 Mio/ml *n* = 15, 4 Mio/ml *n* = 13). The data are expressed as the mean ± SD values. * p < 0.05, ** p < 0.01, **** p < 0.0001, ns no significance.


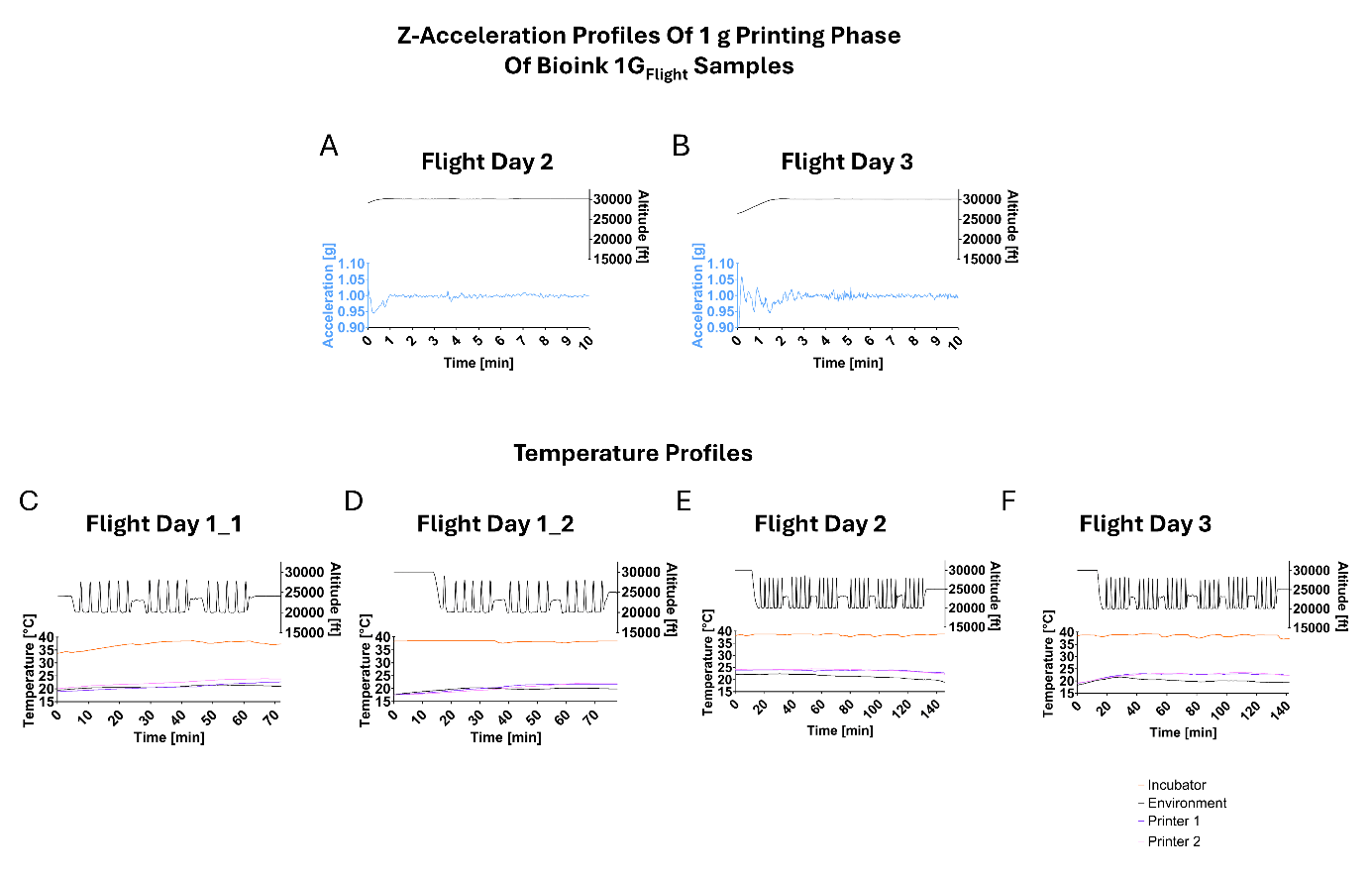


**Figure S4.** Characteristics of In-Flight Environment. Acceleration profile in z-direction [g] and altitude [ft] for flight days 2 (**A**) and 3 (**B**). **C-F** Temperature profile [°C] of the plane environment for each individual flight; inside of the incubator, and of printer 1 and 2 with the correspondent altitude [ft].


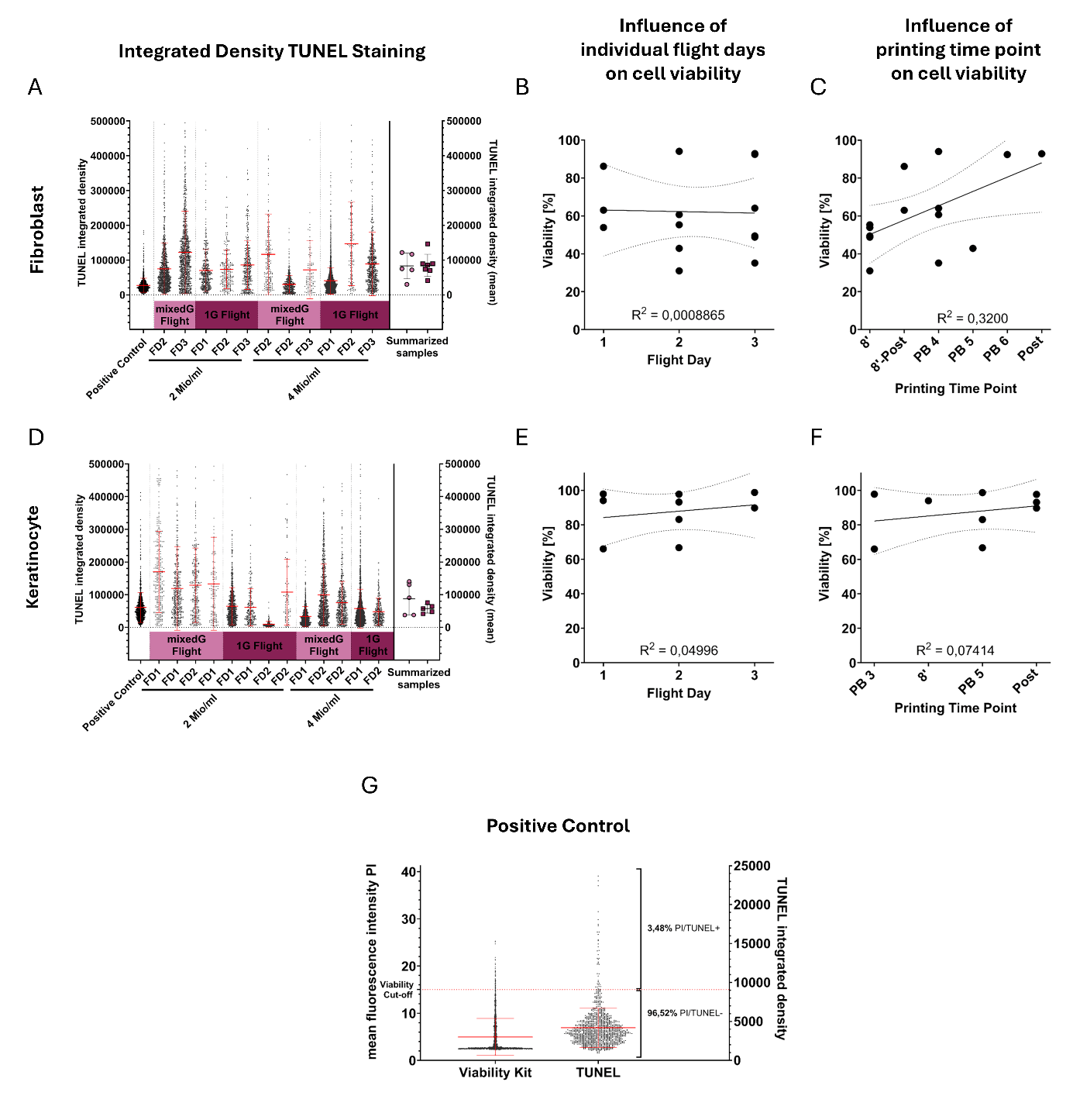


**Figure S5.** TUNEL Cell Viability Staining. **A,D** Integrated density of TUNEL stained hydrogel samples bioprinted in MixedG_Flight_ and 1G_Flight_ throughout the whole campaign; individual samples (left) and summarized results (mean) for respective gravitational condition (right) of the respective cell type (A: fibroblasts, D: keratinocytes). Correlation (Spearman) of cell viability and the respective flight day (**B**,**E**) or the time point of printing (**C**,**F**) of the respective cell type (B,C: fibroblasts, **E**,**F**: keratinocytes) with fitted linear regression curves. Goodness of fit = R^2^. Number of pairs B,C = 14, E,D = 9. **G** Results of viability measurement of positive control. Overall percentage of viable cells determined using Viability Kit (left), data used to define cut-off value for distinguishing viable from non-viable cells based on TUNEL integrated density within the same samples. The data in (A,D,G) are expressed as the mean ± SD values.


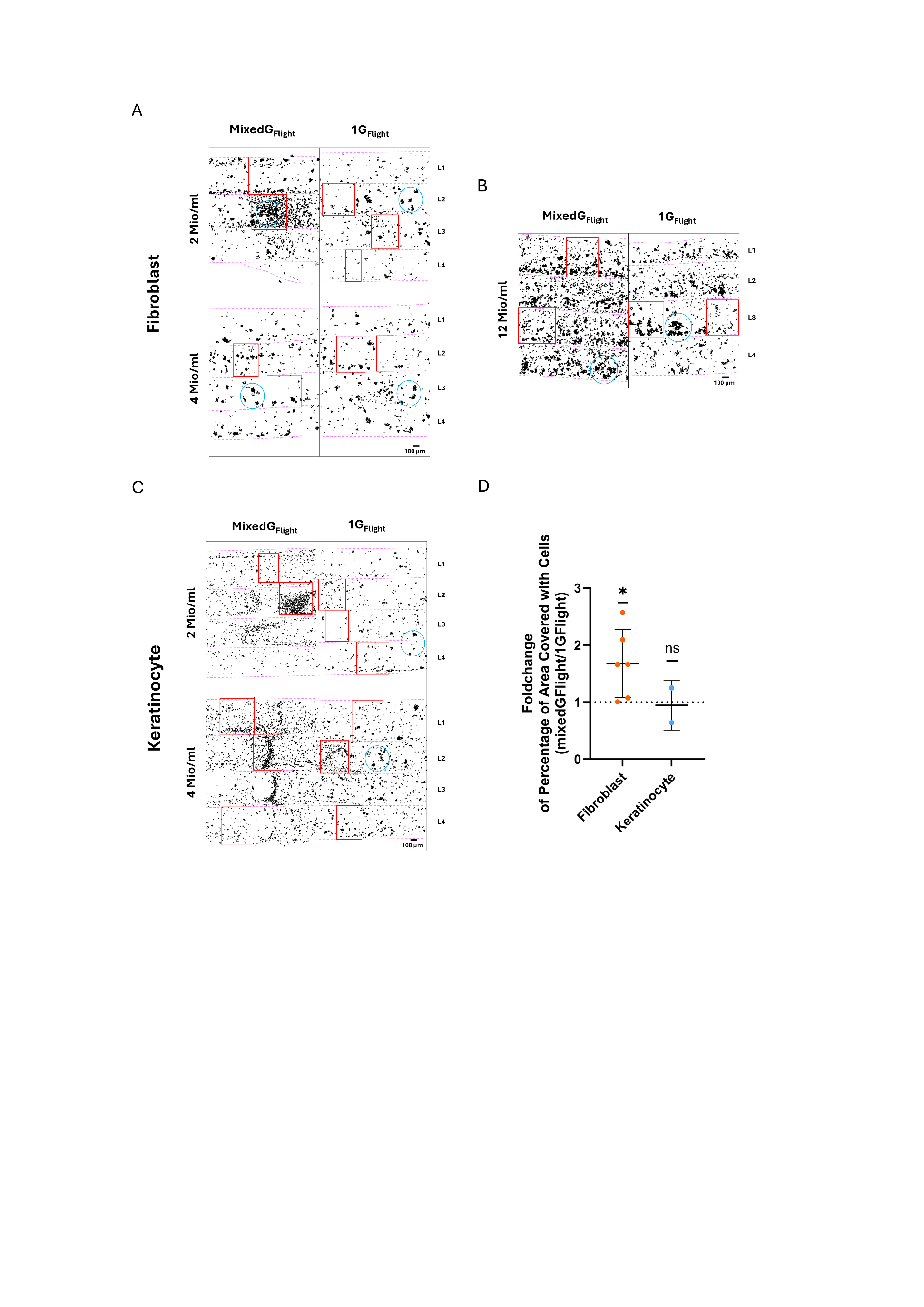


**Figure S6.** Cell accumulation and sedimentation. **A,C** Cell accumulation of fibroblasts (A) and keratinocytes (C) with 2 Mio/ml (top) and 4 Mio/ml (bottom), bioprinted in MixedG_Flight_ (left) and 1G_Flight_ (right) over 105 µm thickness in 4 printed layers (L1-L4) , with exemplary regions of cell accumulation highlighted in red; exemplary regions of cell aggregation highlighted in blue. **B** Cell accumulation of fibroblasts with 12 Mio/ml, same as A; highlighted regions as in A,C. **D** Quantifications of cell accumulation as comparison between the gravitational conditions. Total area covered with cells inside of each hydrogel was determined and the ratio to the total hydrogel area calculated; the foldchange between the respective hydrogel bioprinted in MixedG_Flight_ and 1G_Flight_ with fibroblasts (orange) and keratinocytes (blue) with 2 Mio/ml or 4 Mio/ml was calculated (105 µm thickness). Statistical analysis between the foldchange values and the value 1.0 (no difference). Statistical comparisons were performed via one sample t-test (Fibroblast) and Wilcoxon signed-rank test (Keratinocyte). Number of replicates: Fibroblast n = 6, Keratinocyte n = 2. The data are expressed as the mean values with SD. * p < 0.05, ** p < 0.01, **** p< 0.0001, ns no significance. Scale bar = 100 µm.


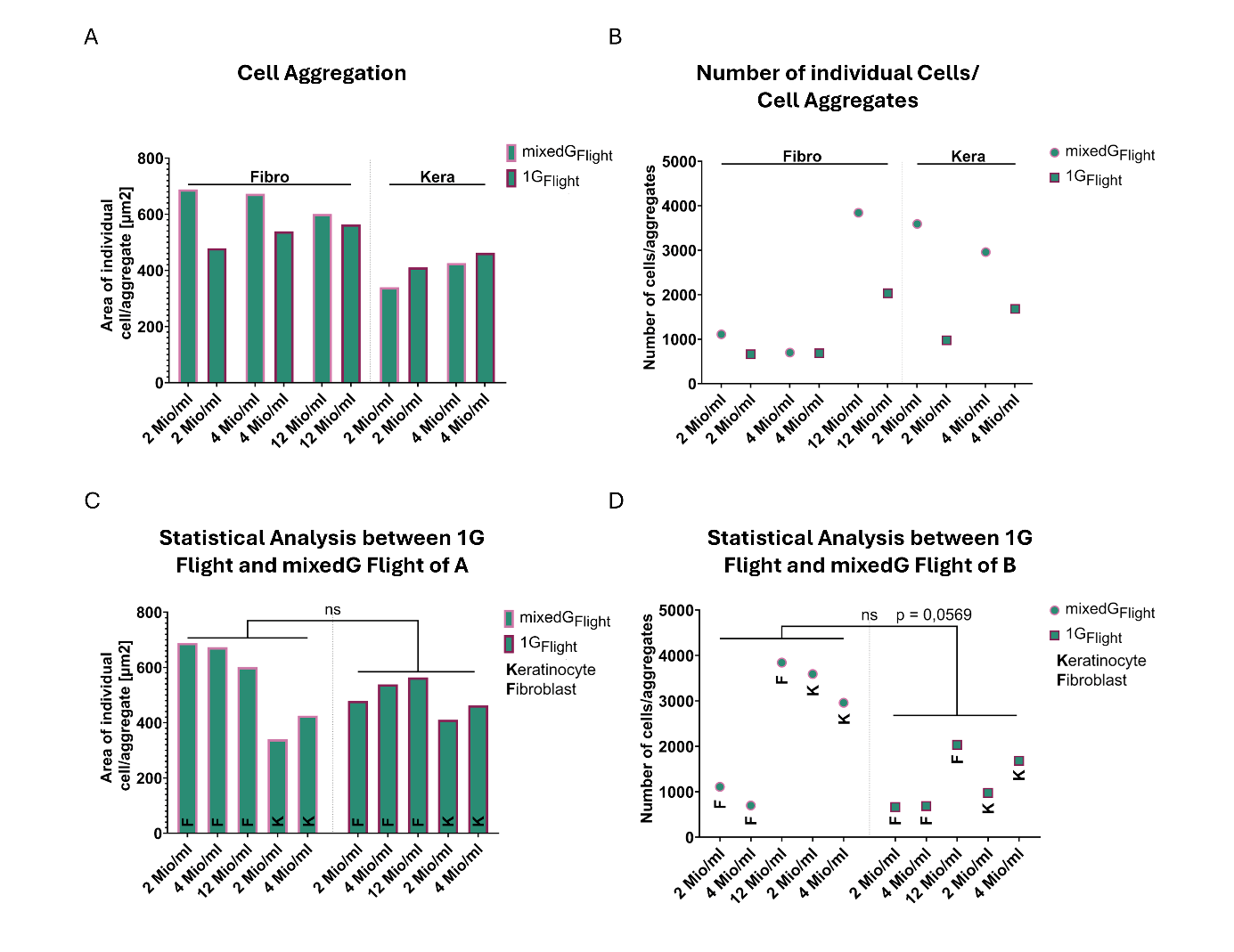


**Figure S7.** Cell Aggregation. Area (**A**) and total number (**B**) of individual cells/cell aggregates for fibroblasts and keratinocytes in MixedG_Flight_ and 1G_Flight_ with cell concentrations of 2, 4 and 12 Mio/ml. **C**,**D** Statistical analysis between MixedG_Flight_ and 1G_Flight_ conditions of A and B. Statistical comparisons were performed via paired t-test. Number of replicates: MixedG_Flight_ *n* = 5, 1G_Flight_ *n* = 5. The data in (A,C) are expressed as the median values, in (B,D) as the mean value. * p < 0.05, ** p < 0.01, **** p < 0.0001, ns no significance.

**Table S1.** Absolute values (Mean with SD) of hydrogel dimensions (width) and number of replicates for hydrogels printed with the respective ink under the respective conditions.

| **Photoink** | | | | |
| --- | --- | --- | --- | --- |
| **Target width  (in CAD) [µm]** | 1G_Ground_ | | MixedG_Flight_ | |
|  | absolute size [µm] | n-number | absolute size [µm] | n-number |
| **300** | 269.1 ± 32.57 | 12 | 357.6 ± 33.06 | 3 |
| **400** | 303 ± 50.45 | 15 | 382.8 ± 46.26 | 5 |
| **500** | 388.6 ± 47.23 | 15 | 435.2 ± 37.91 | 6 |
| **600** | 508.3 ± 40.8 | 15 | 491.4 ± 59.49 | 6 |
| **700** | 616.1 ± 46.83 | 15 | 563 ± 33.54 | 6 |
| **800** | 588.9 ± 86.11 | 15 | 586.4 ± 59.47 | 6 |
| **900** | 764.8 ± 76.71 | 15 | 765.8 ± 47.04 | 9 |
| **1000** | 843.4 ± 68.87 | 15 | 854 ± 100.2 | 5 |
| **2000** | 1564 ± 60.80 | 10 | 1568 ± 106 | 5 |
| **3000** | 2493 ± 112.8 | 10 | 2650 ± 191.6 | 6 |

| **Photoink** | | | | |
| --- | --- | --- | --- | --- |
| **Target width  (in CAD) [µm]** | MixedG_Flight_ | | 1G_Flight_ | |
|  | absolute size [µm] | n-number | absolute size [µm] | n-number |
| **200** | 314.2 ± 62.99 | 7 | 299.9 ± 62.4 | 5 |
| **300** | 398.3 ± 77.3 | 9 | 421.5 ± 69.28 | 12 |
| **400** | 464.8 ± 102.1 | 11 | 537.7 ± 78.51 | 12 |
| **500** | 431.2 ± 51.8 | 10 | 523.2 ± 106.6 | 12 |
| **600** | 512.3 ± 36.80 | 11 | 574.6 ± 139.3 | 12 |
| **700** | 578.9 ± 47.44 | 11 | 646.4 ± 87.12 | 12 |
| **800** | 682.8 ± 43.81 | 9 | 691.8 ± 138.7 | 12 |
| **900** | 753.3 ± 45.42 | 12 | 797.4 ± 148.1 | 10 |

| **Bioink With Cells** | | | | | | |
| --- | --- | --- | --- | --- | --- | --- |
| **Target width  (in CAD) [µm]** | 1G_Ground_ | | MixedG_Flight_ | | 1G_Flight_ | |
|  | absolute size [µm] | n-number | absolute size [µm] | n-number | absolute size [µm] | n-number |
| **2000** | 1721 ± 55.99 | 18 | 1726 ± 51.22 | 28 | 1721 ± 55.99 | 18 |
| **4000** | 3423 ± 113.8 | 18 | 3439 ± 84.5 | 28 | 3423 ± 113.8 | 18 |
| **6000** | 5074 ± 165.4 | 18 | 5096 ± 99.15 | 28 | 5074 ± 165.4 | 18 |

**Table S2.** Percentage (Mean with SD) of hydrogel deviation from target dimension and number of replicates for hydrogels printed with the respective ink under the respective conditions.

| **Photoink** | | | | |
| --- | --- | --- | --- | --- |
| **Target width  (in CAD) [µm]** | 1G_Ground_ | | MixedG_Flight_ | |
|  | Deviation from target dimension [%] | n-number | Deviation from target dimension [%] | n-number |
| **300** | -10.31 ± 10.86 | 12 | 19.22 ± 11.02 | 3 |
| **400** | -22.05 ± 13.16 | 15 | -4.289 ± 11.57 | 5 |
| **500** | -18.73 ± 6.547 | 15 | -12.97 ± 7.582 | 6 |
| **600** | -13.18 ± 5.175 | 15 | -18.10 ± 9.916 | 6 |
| **700** | -9.625 ± 5.033 | 15 | -19.57 ± 4.792 | 6 |
| **800** | -22.75 ± 7.763 | 15 | -26.70 ± 7.433 | 6 |
| **900** | -13.05 ± 8.426 | 15 | -14.91 ± 5.227 | 9 |
| **1000** | -14.79 ± 6.899 | 15 | -14.6 ± 10.02 | 5 |
| **2000** | -21.68 ± 3.37 | 10 | -21.59 ± 5.301 | 5 |
| **3000** | -15.91 ± 2.842 | 10 | -11.67 ± 6.388 | 6 |

| **Photoink** | | | | |
| --- | --- | --- | --- | --- |
| **Target width  (in CAD) [µm]** | MixedG_Flight_ | | 1G_Flight_ | |
|  | Deviation from target dimension [%] | n-number | Deviation from target dimension [%] | n-number |
| **200** | 57.09 ± 31.5 | 7 | 49.97 ± 31.2 | 5 |
| **300** | 32.76 ± 25.77 | 9 | 40.51 ± 23.09 | 12 |
| **400** | 16.2 ± 25.52 | 11 | 34.41 ± 19.63 | 12 |
| **500** | -13.77 ± 10.36 | 10 | 4.636 ± 21.33 | 12 |
| **600** | -14.61 ± 6.134 | 11 | -4.229 ± 23.22 | 12 |
| **700** | -17.3 ± 6.777 | 11 | -7.663 ± 12.45 | 12 |
| **800** | -14.66 ± 5.480 | 9 | -13.53 ± 17.33 | 12 |
| **900** | -16.3 ± 5.047 | 12 | -11.41 ± 16.45 | 10 |

| **Bioink With Cells** | | | | | | |
| --- | --- | --- | --- | --- | --- | --- |
| **Target width  (in CAD) [µm]** | 1G_Ground_ | | MixedG_Flight_ | | 1G_Flight_ | |
|  | Deviation from target dimension [%] | n-number | Deviation from target dimension [%] | n-number | Deviation from target dimension [%] | n-number |
| **2000** | -13.97 ± 2.8 | 18 | -13.71 ± 2.561 | 28 | -13.97 ± 2.8 | 18 |
| **4000** | -14.43 ± 2.844 | 18 | -14.03 ± 2.113 | 28 | -14.43 ± 2.844 | 18 |
| **6000** | -15.43 ± 2.757 | 18 | -15.07 ± 1.652 | 28 | -15.43 ± 2.757 | 18 |
